# Salts and Polymorph Screens for Bedaquiline

**DOI:** 10.1208/s12249-021-02106-7

**Published:** 2021-08-25

**Authors:** Mercy Okezue, Susan Bogdanowich-Knipp, Daniel Smith, Matthias Zeller, Stephen Byrn, Pamela Smith, Dale K. Purcell, Kari Clase

**Affiliations:** 1 Biotechnology Innovation and Regulatory Science Center, ABE, Purdue University, 225 S. University Street, Indiana 47906 West Lafayette, USA; 2 Ravine Pharmaceuticals, LLC, 3425 DuBois St., Indiana West Lafayette, USA; 3 Industrial and Physical Pharmacy, Purdue University, 575 Stadium Mall, Indiana 47907 West Lafayette, USA; 4 Chemistry, Purdue University, 560 Oval Dr, Indiana 47907-2084 West Lafayette, USA; 5 Improved Pharma LLC, 1281 Win Hentschel Blvd, Indiana 47906 West Lafayette, USA; 6 Leading With Smart Science, LLC, 5315 Shootingstar Ln, Indiana West Lafayette, USA; 7 Chemical Microscopy LLC, 1281 Win Hentschel Blvd, Indiana 47906 West Lafayette, USA

**Keywords:** Bedaquiline, Salt screen, Polymorph screen, X-ray diffraction and Tuberculosis

## Abstract

Bedaquiline is used to treat multi-resistant tuberculosis in adults. The fumarate salt is commercially available and used in the product Sirturo. To provide open access to bedaquiline molecule once the patent on the chemical substance expires, new salts were screened. This work offers additional information on the bedaquiline system, as new salts may present better pharmacokinetic properties. The current studies focus on the attempted isolation of the acetate, benzoate, benzenesulfonate, hydrobromide, succinate, hydrochloride, tartrate, lactate, maleate, malate, and mesylate salts of bedaquiline. Potential salts were screened using a unique combination of conventional screening, and small-scale experiments supplemented by crystallographic analysis and infrared microspectroscopy. Salts were prepared on a larger scale by dissolving 1:1 ratios of the individual salt formers and bedaquiline base (30 mg, 0.055 mmol) in different solvents and allowing the solutions to evaporate or crystallize. X-ray diffraction (XRD) techniques and spectroscopic and thermal analyses were employed to characterize the salts. The benzoate and maleate salts were selected as lead candidates after reviewing preliminary characterization data. To determine the most stable forms for the leads, a polymorph screen was conducted using solvents of various polarities. These salt screens successfully generated five new salts of bedaquiline, namely, benzoate, maleate, hydrochloride, besylate, and mesylate. The existence of these salts was confirmed by powder XRD, proton NMR, and IR spectroscopies. TGA and DSC thermal analysis along with hot-stage optical microscopy were further used to characterize the salts. The polymorph screen conducted on the salts suggested the absence of additional polymorphs at 1 g scale.

**Supplementary Information:**

The online version contains supplementary material available at 10.1208/s12249-021-02106-7.

## INTRODUCTION

The solid-state form of pharmaceuticals impacts physicochemical properties during drug development, so, it is important that a manufacturer understands the material properties of the API and the finished product. The ICH Q6 document (1), published by the International Council for Harmonization of Technical Requirements for Pharmaceuticals for Human Use, provides guidance for generating specifications for new drug substances and new drug products. However, time and material constraints may hinder a full analysis of the solid-state properties of an API in the early stages of drug development (2). In such instances, a drug developer can conduct some simple screening and provide an abbreviated solid-state chemistry profile for the new material. During the screening process in early drug development, it is beneficial to identify the most stable drug form before scaling up production of the material, as the API form selection impacts solubility, bioavailability, stability, and other physicochemical properties. It is therefore important to know the relative stabilities of the various polymorphic forms of the drug before scale-up of drug production, to reduce the risk of transformation that may occur during the non-clinical and clinical stages (3).

Bedaquiline, (alpha S. beta R)-6-bromo-alpha-2-(dimethylamino)ethyl-2-meth oxy-alpha-1-naphthalenyl-beta-phenyl-3-quinolineethanol, was approved by the US FDA in 2012 to treat multi-resistant pulmonary tuberculosis in adults (4). The drug showed efficacy and achieved the surrogate endpoint of converting positive mycobacterium tuberculosis sputum to negative results in double-blinded phase 2 clinical trials (5). Bedaquiline fumarate is marketed as tablets under the brand name Sirturo by Janssen Pharmaceuticals (6, 7); the patent (US 8,546,428) for the fumarate salt was filed by Hegyi *et al.* in 2013 (8). Zvatora and colleagues from the Czech Republic acquired WIPO patents for the sulphate, tartrate, citrate, and phosphate salts of bedaquiline (9, 10). A doctoral dissertation used a crystal engineering strategy to identify BDQF-NAC, a novel co-crystal of bedaquiline fumarate (11). Salt screening experiments, currently underway in our laboratories, focused on the isolation of acetate, benzoate, benzenesulfonate, hydrobromide, succinate, hydrochloride, tartrate, lactate, maleate, malate, and mesylate salts of bedaquiline. The studies showed that the benzoate salts could be obtained in at least two forms: as a stable 1.17-hydrate obtained from, e.g., acetone and propanol, and a second form, an acetonitrile solvate, in which the partially occupied water molecule is replaced by a 74.2% (7) occupied acetonitrile molecule (12). Likewise, the maleate salts existed as a hemi-hydrate from acetone or as a THF solvate with an occupancy of 2 mol THF per bedaquiline maleate molecule (manuscript in preparation). The overall goal of these studies was to find salts (and their potential polymorphs) not claimed in patents to provide open access to the bedaquiline molecule once the patent on the chemical substance expired.

### Solid-State Forms

Drug substances used in pharmaceutical formulations can exist in various forms. The molecules in crystalline materials usually pack in an orderly arrangement, while amorphous materials display short-range disordered packing. The presence of three-dimensional translational symmetry in crystalline materials gives them a characteristic X-ray diffraction pattern. Amorphous materials do not give a diffraction pattern, though materials can show intermediate degrees of order or contain mixtures of amorphous and crystalline forms (13).

Crystals of a pharmaceutical material may exhibit different morphologies, or habits. The variations in shapes and sizes may cause these crystals to occur as needles, plates, blades, and rods. Different crystal habits can occur even for an otherwise identical material with the same microscopic crystal structure, but a different habit can also be an indication that a different polymorph or solvate might have formed. Polymorphs are crystals that have the same chemical structure but have different molecular packing or internal structure (13, 14). Solvates are crystals that have molecules of solvents regularly incorporated within the lattice of the molecule (15) and are sometimes referred to as “pseudopolymorphs.” Thus, when a pharmaceutical material exhibits different crystal habits, further evaluation needs to be conducted to determine if they are polymorphs or solvates or have the same crystal packing arrangements (13). Polymorphs of the same API may exhibit different solubilities, so it is important to identify how to control their formation in pharmaceutical formulations (16, 17). Regardless of the shapes (habits), X-ray powder diffraction from the same polymorph will typically yield the same or nearly the same diffraction pattern (13).

### Decision Trees in Pharmaceutical Development

It is important to identify and characterize possible forms of a pharmaceutical molecule before getting to the critical scale up and optimization stages. A group of reviewers also suggested use of solid-state chemistry in early drug discovery to increase the prospects of identifying not only the most stable, but also the most developable form with optimal *in vivo* characteristics (18). The US FDA together with Byrn and coworkers published a report that provides guidance on how decision trees can be used in early stages of pharmaceutical development to reduce risk and design formulations (19). Similar decision trees are provided in the ICH Q6 document. When different polymorphs of a molecule are identified, then it is necessary to further characterize them. Physical tests such as solubility, melting points and stability determinations help predict if the observed differences will impact drug product quality and performance (1).

The provisions for specifications in ICH Q6A describe universal and specific tests and criteria recommended for new drug substances and finished products. Specifications are lists of tests, references to the analytical procedures to conduct the tests listed, together with acceptance criteria, which specify limits of acceptance for the test results (1). The universal test for new drug substances includes description, identification, assay, and impurities. The specific tests comprise physicochemical properties (e.g., pH, melting point, and refractive index), particle size, polymorphic forms, chiral tests, water content, inorganic impurities, and microbial limits.

Polymorphism of a new drug substance describes existence of crystalline forms which differ in their physical properties. In a regulatory environment, the term also covers solvation or hydration (pseudopolymorphs), as well as amorphous forms of a new moiety (1, 20, 21). The appropriate solid state of an API should be specified when there is evidence of potential differences in performance, bioavailability, or stability of the drug product.

Physicochemical measurements that can identify the existence of multiple forms includes melting point measurements (including hot-stage microscopy), X-ray diffraction (XRD), thermal analysis procedures (differential scanning calorimetry (DSC), thermal gravimetric analysis (TGA), and DTA), infrared, and spectroscopy, optical microscopy, and solid-state NMR (1). The current study utilized some of these techniques and provides results obtained from the salt screen and analysis of newsalts of bedaquiline.

### Polymorph Screening in Early Phase Drug Development

During early phase of development, it is important to discover the most thermodynamically stable polymorphic form(s) of a drug, that is the most stable under ambient conditions. This desirability is borne out of the consideration that the most stable form will have the least probability to undergo transformation during scaleup, manufacturing, and storage (22). Solid-state determinations can usually be done in the preformulation and formulation design stages of drug development to discover the existence of a form with fast dissolution kinetics that will yield adequate bioavailability. Therefore, the identified polymorphs of a crystalline material are screened to determine the form that will yield the most desirable form for development. It is advisable to include solvents used in the last crystallization steps and manufacturing (13). Polymorph screening can be achieved by recrystallization from a wide range of solvents, using techniques like heating/cooling, antisolvent addition, and fast and slow evaporation (22, 23). Solvents with varying dielectric constants can be employed (22). However, an alternative strategy selects solvents based on customized interaction profile maps developed from virtual supersaturated solution simulations of API-solvent interactions (24). Also, other efforts suggested the rational use of computational approaches designed to predict conformational populations in solutions for crystallizing polymorphs (25). In this study, the selection was based on properties such as polarity and hydrogen bonding propensity, so solvents of varying dielectric constant were used.

### Study Objectives

1. Carry out a standard screen to generate salts of bedaquiline.
2. Prepare additional quantities of crystalline materials and partially characterize them.
3. Conduct a polymorph screen on selected crystalline salts discovered.

## MATERIALS AND METHOD

### Recovery of Bedaquiline Base from the Fumarate Salt

At the start of the experiment only the fumarate salt was available. The free base was recovered from the commercially available fumarate salt by extracting a dichloromethane (CH_2_Cl_2_) solution three times with saturated sodium bicarbonate (NaHCO_3_) solution (12, 26). The recovered base was evaporated over 24 h to remove excess solvents. The residue crystallized giving crystals of the free base. The crystal structure of this material is reported in a previous paper (12).

### Conventional Salt Screen Experiments

The conventional method of generating salts through acid–base reactions was applied. Ten acids (salt formers) were investigated in the attempt to synthesize the corresponding salts of bedaquiline base: acetate, benzenesulfonate, benzoate, hydrobromide, hydrochloride, lactate, fumarate, maleate, malate, mesylate, and succinate. Bedaqiline is poorly soluble in water and many polar solvents (6). Therefore, the approximate volumes of some ICH classes 2 and 3 solvents required to dissolve ~ 30 mg free base was the criteria for solvent selection. Acetone (< 1 mL), acetonitrile (~ 5 mL), 2-propanol (~ 5 mL), and tetrahydrofuran (< 1 mL), provided the lowest volumes for dissolving the free base. Potential salts were screened by dissolving 1:1 ratios of the individual salt formers and bedaquiline base (30 mg, 0.055 mmol) in different solvents. For the dicarboxylic acids, succinic, maleic and malic acids, 1:1 and 1:2 ratio experiments were conducted. The equivalent amounts of acids used in the various experiments are shown in Table I. Details of all reagents used in the salt screen is provided in the Electronic Supplementary Materials (ESM), Table XIV.

**Table I:** Acids (salt formers) and equivalent weights used in synthesis

Acid (salt former) used	Acid (weight, mg) equivalent to 30 mg bedaquiline base (0.054 mmol)
Acetic acid	3.24
Benzenesulfonic acid	8.54
Benzoic acid	6.60
Hydrobromic acid aq	4.37
Hydrochloric acid aq	1.97
Lactic acid (hydroxypropanoic acid)	4.86
Maleic acid	6.27
Malic acid	7.24
Methanesulfonic acid (methylsulfonic acid)	5.19
Succinic acid (butanedionic acid)	6.38
Fumaric acid	6.27

The solutions obtained were exposed to various experimental conditions. Slow and fast evaporation were easier to set up, but some of the experiments yielded amorphous solids. So, other methods of forming crystalline salts were explored, including: heating followed by evaporation, and solvent/antisolvent addition using water and/or hexanes as the antisolvent. Procedures for sample preparation and the experimental conditions are shown in Table II.

**Table II:** Procedures for sample preparations for salt screening

Steps	Evaporation experiments	Notes
1	Prepared 10 mL of solution of BQ containing 30 mg/mL of each solvent	This produced volumetrics with 10 mL BQ each solvent
2	Prepared 10 mL of solution of each of 10 salt formers containing approximately 30 mg/mL	This produced volumetrics with 10 mL of each salt former in each solvent
3	Placed 1 mL of BQ solution (contains 30 mg BQ) in a scintillation vial	This produced scintillation vials with 1 mL BQ each for different solvents
4	Added stoichiometric amount of salt former solution to vials containing 1 mL of BQ	Produced vials containing BQ and 1 of 10 salt formers in different solvent
5	Allowed the vials to evaporate (fast evaporation)	Analyzed solids produced by X-ray diffraction and/or microscopy
6	Inserted pin holes in foil aluminum cap covering vial	Analyzed solids produced by X-ray diffraction and/or microscopy
7	Used water as antisolvent to crystallize salt from solution	Analyzed solids produced by X-ray diffraction and/or microscopy

### Screen for Stable Polymorph

Polymorph screening was conducted with solvents of varying polarity (Table III) and with various solvent systems that might encourage new forms or be used in the production process. About 15 mg to 20 mg of the identified crystalline salts were subjected to heating and cooling, fast and slow evaporation, solvent/antisolvent precipitation, vapor diffusion, liquid/liquid diffusion, and 1-week slurring experiments. About 92 experiments were conducted on the benzoate and maleate salts in efforts to identify possible polymorphic forms.

**Table III:** Summary of solvents used in polymorph identification screen

Solvent	Dielectric constant @ 20 °C (a measure of solvent polarity)
Hexanes	1.88
Ethyl acetate	6.02
Tetrahydrofuran (THF)	7.6
Trifluoroethanol (TFE)	8.55
2-Propanol	20.18
Acetone	20.7
Propyl alcohol	21.8
Ethanol	25.3
Methanol	33.0
Dimethylformamide (DMF)	36.71
Acetonitrile	37.5
Water	80.1

### Powder X-ray Diffraction Determinations and Refinement Data

XRD was performed on a Panalytical Empyrean Powder X-ray Diffractometer fitted with Bragg–Brentano HD optics, a sealed tube copper X-ray source (*λ* = 1.54178 Å), and a PixCel3D Medipix detector. The X-ray tube with a copper anode was operated at 45 kV and 40 mA. Dried samples were pulverized using an agate mortar and pestle and packed into a zero-background single crystal silicon sample holder, 16 mm wide and 0.25 mm deep. Each sample was measured for 30 min (5 scans at 6 min each) with a fixed mask of 4 mm, an antiscatter slit of 1/4°, and a divergence slit of 1/16°. The scattered intensities were measured over a 2θ angular domain from 4° to 40° θ at ambient conditions using the Panalytical Data Collector software (27).

Search/Match phase identification was performed using the HighScore software of Panalytical against the ICDD PDF4/Organics database (28). Rietveld refinements were performed against the models of the single crystal structure data sets using the HighScore software of Panalytical (29). If required, refinement of preferred orientation was included using a spherical harmonics model.

### Single Crystal X-ray Structure Determinations

One of the important strategies for this study involved hand isolating using microscopy or the naked eye, crystals that formed from the various evaporations. Even if a complex mixture of salts and solvates was formed by crystallization, single crystal determination provided a direct confirmation of one of the species present. In some cases, this was a desirable salt and additional experiments could be performed to develop an optimum method for isolating the salt.

In some cases, this method of manual isolation yielded the free base which was presumably formed by some type of disproportionation process. This information was also useful in that it indicated that in this system salt disproportionation was a potential challenge.

Once single crystals were extracted from the mixture of solids they were coated with a trace of Fomblin oil and transferred to the goniometer head of one of two instruments: either a Bruker Quest diffractometer with a fixed chi angle, a Mo Kα wavelength (*λ* = 0.71073 Å) sealed tube fine focus X-ray tube, single crystal curved graphite incident beam monochromator, and a Photon 100 or Photon II area detector. Or a Bruker Quest diffractometer with kappa geometry, a Cu Kα wavelength (*λ* = 1.54178 Å) I-μ-S microsource X-ray tube, laterally graded multilayer (Goebel) mirror for monochromatization, and a Photon II or Photon III C14 area detector. Both instruments were equipped with an Oxford Cryosystems low-temperature device, and examination and data collection were performed at 150 K. Data were collected, reflections were indexed and processed, and the files scaled and corrected for absorption using APEX3(30, 31) and SADABS. The space groups were assigned using XPREP within the SHELXTL suite of programs (32) and solved by direct methods using ShelXS (33) and refined by full matrix least squares against *F*^2^ with all reflections using Shelxl2018 using the graphical interface Shelxle (34, 35). H atoms attached to carbon and nitrogen atoms as well as hydroxyl hydrogens were positioned geometrically and constrained to ride on their parent atoms. C–H bond distances were constrained to 0.95 Å for aromatic and alkene C–H moieties, and to 1.00, 0.99, and 0.98 Å for aliphatic C-H, CH_2_ and CH_3_ moieties, respectively. N–H bond distances were constrained to 0.88 Å for planar (sp^2^ hybridized) N–H and N–H^+^ groups. O–H distances of alcohols were constrained to 0.84 Å. Methyl CH_3_, ammonium NH_3_^+^, and hydroxyl H atoms were allowed to rotate but not to tip to best fit the experimental electron density. Water H atom positions were refined, and O–H distances were restrained to 0.84(2) Å. Where necessary, water H⋅⋅⋅H distances were restrained to 1.36(2) Å, and H atom positions were further restrained based on hydrogen bonding considerations. U_iso_(H) values were set to a multiple of U_eq_(C) with 1.5 for CH_3_ and OH, and 1.2 for C–H, CH_2_, N–H, and NH_2_ units, respectively.

### Melting Point

Melting points of the crystalline salts were determined using a Thomas Hoover Capillary Melting Point apparatus, and values were uncorrected.

### Differential Scanning Calorimetry

DSC analyses were performed on a TA Instruments Q10 differential scanning calorimeter, equipped with a refrigerated cooling system (RCS 90). The system was calibrated for both temperature and enthalpy using an indium standard. Samples were weighed into standard aluminum pans and covered with a lid containing a pin hole, crimped, and placed onto the sample side of the thermoelectric disk inside the furnace. An empty pan with the same configuration as the sample was placed onto the reference side of the thermoelectric disk inside the furnace. Samples were heated from 25 °C to a maximum of 300 °C at 10 °C/min using a nitrogen purge of 45–50 psi.

### Thermal Gravimetric Analysis

TG analyses were performed using a Mettler-Toledo TGA/DSC3 + analyzer. Temperature and enthalpy adjustments were performed using indium, tin, zinc, and phenyl salicylate, and then verified with indium. The balance was verified with calcium oxalate. The samples were placed in aluminum pans, hermetically sealed, the lids pierced, and then inserted into the TG furnace. A weighed aluminum pan configured as the sample pan was placed on the reference platform. The data acquisition parameters for the thermograms are displayed in the images. The method name on the thermograms is an abbreviation for the start and end temperature as well as the heating rate: e.g., “25–350-10” means from ambient to 350 °C, at 10 °C/min.

### Water Content by Karl Fischer Titration

The water content for the benzoate salts were conducted using a Metrohm 831 KF coulometer, fitted to a 703 Ti stand for the titration vessel. The solvent used was Hydranal Coulomat AG manufactured by Honeywell Fluka. The weights of samples titrated were about 50 mg and 30 mg of the benzoate salts from 2-propanol-water and acetone solvents, respectively.

### Hygroscopicity

Hygroscopicity was monitored by exposing select samples to either 75% RH or 0% RH conditions for a minimum of 1 week. Small chambers were set up containing either a saturated sodium chloride solution (75% RH) or Drierite (0%RH) (36). Samples were weighed into a pre-weighed vial and cap, the sample weight recorded, and the vial placed uncapped inside of the appropriate chamber. The chamber was tightly closed and allowed to sit at ambient temperature. At select time intervals, each vial containing sample was removed from the chamber, capped, and weighed. The amount of weight gain or loss was then calculated based upon the initial sample weight prior to exposure to the condition (37).

### Nuclear Magnetic Resonance

Nuclear magnetic resonance (NMR) data were collected in acetonitrile-d3 (ACN-d3) using a Bruker DRX-500 spectrometer and were referenced against the residual nondeuterated solvent peak.

### Microscopy (Stereo and Polarized Light Microscopy)

A small portion of sample was placed on a cleaned microscope slide and a no. 1 ½ cover glass placed over the sample. Mineral oil, USP, (CAS: 8042–47-5) was allowed to cover the sample by capillarity. Images were acquired as a collection of three: (1) plane-polarized light, (2) cross-polarized light, and (3) cross-polarized light with first-order red compensator. Microscopical observations revealed crystal habit as birefringent, platy, anhedral agglomerates that are softly bound and easily dispersed under light pressure from a tungsten needle on the cover glass.

### Hot-Stage Optical Microscopy

A small portion of sample was placed on a cleaned microscope slide and a no. 1 ½ cover glass placed over the sample. In oil preparations, either mineral oil, USP (CAS: 8042–47-5) or high temperature, silicone oil (CAS: 68083–14-7) was allowed to cover the sample by capillarity. The system calibration was verified with melting point standards prior to analyses.

Analyses were completed using an Olympus BX51TRF polarized light microscope using crossed-polarizers, a 20 × , 0.40 Numerical Aperture, LM PLAN FL N objective, and a first-order red compensator. Heating was conducted with a Linkam LTS420 hot stage with a T95 LinkPad system controller. Images were acquired using a Lumenera Infinity-X color digital camera and Infinity Analyze v.6.5.5 (build 2016). The Linkam hot stage system controller was programmed with a ramp routine as follows: (1) heating at 10 °C/min to a minimum of 85 °C, (2) heating at 5 °C/min to a minimum of 120 °C, and (3) heating at 2 °C/min to a minimum of 190 °C. (This ramp is sample/test specific and my change per individual sample.)

### Fourier Transform Infrared Microspectroscopy

Infrared analyses were performed by reflection/absorption (R/A) using an all-reflecting objective (ARO), 15 × , 0.88 NA. A small amount of sample was transferred to a low-E microscope slide (Smiths Detection P/N: 006–4013) and dispersed to a thin layer. Infrared microprobe analyses were conducted on what appeared microscopically to be single a crystal. Additionally, infrared spectra were collected by attenuated total reflection (ATR) using a type IIIa hemispherical diamond internal reflection element (IRE), dATR objective, 36 × , 0.88 NA. The dATR was brought into contact with what appeared microscopically to be a single crystal. The single crystal may be observed during analyses to ensure on which area of the sample analyses is being conducted, which is observed as “wetting” of the diamond surface as appropriate pressure is applied. A Fourier transform infrared microspectroscopy (FT-IR) spectral background was collected immediately prior to each sample spectral analysis.

## RESULTS

### PXRD and Single Crystal X-ray Diffraction Data from Salt Screen Experiments

**Fig. 1.**
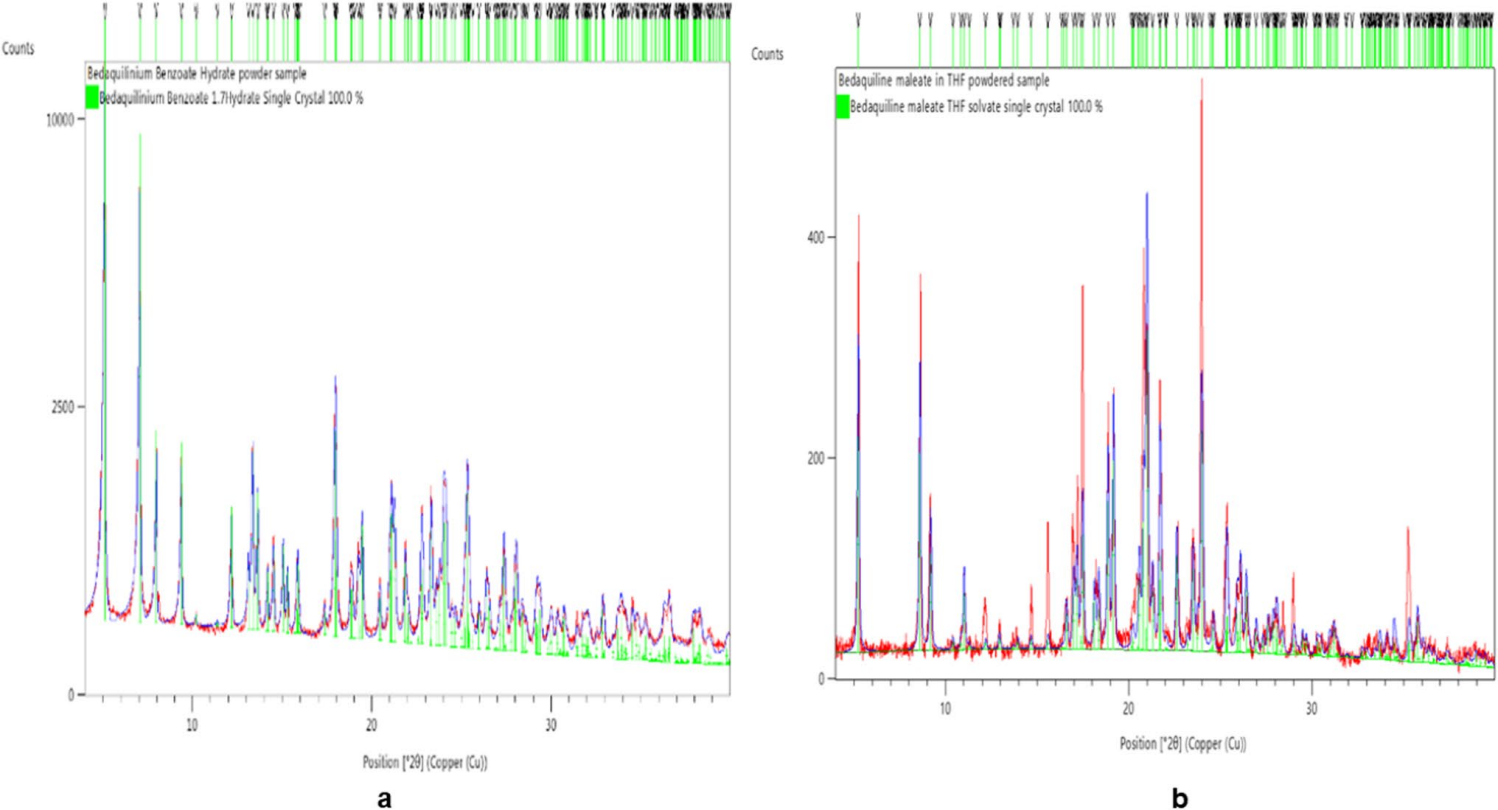
**a** PXRD from benzoate from acetone experiment gave a good fit when refined against the bedaquiline hydrate single crystal. **b** PXRD from maleic acid: bedaquiline, slow and fast evaporation in THF gave a good fit when refined against bedaquiline maleate THF single crystal.

Crystalline salts of bedaquiline were formed from the 1:1 stoichiometric acid–base mixtures from some counterions, while others did yielded amorphous materials, or did not form any precipitates. Summary of the results for the experiment are provided as Tables XV, XVI, XVII, and XVIII in the ESM. Rietveld refinement of the PXRD for benzoate and maleate salts of bedaquiline are shown below (Fig. 1). The diffractograms for other benzoates (Fig. VII), hydrochloride and besylate (Fig. VIII), mesylate, and lactate (Fig. IX), salts are found in the ESM section. Two overlays of the salts PXRD is provided as Figs. XX and XXI in the ESM. PXRD of all the salts showed identifiable diffraction patterns at 2-theta angles that differentiates them from the free base.

**Fig. 2.**
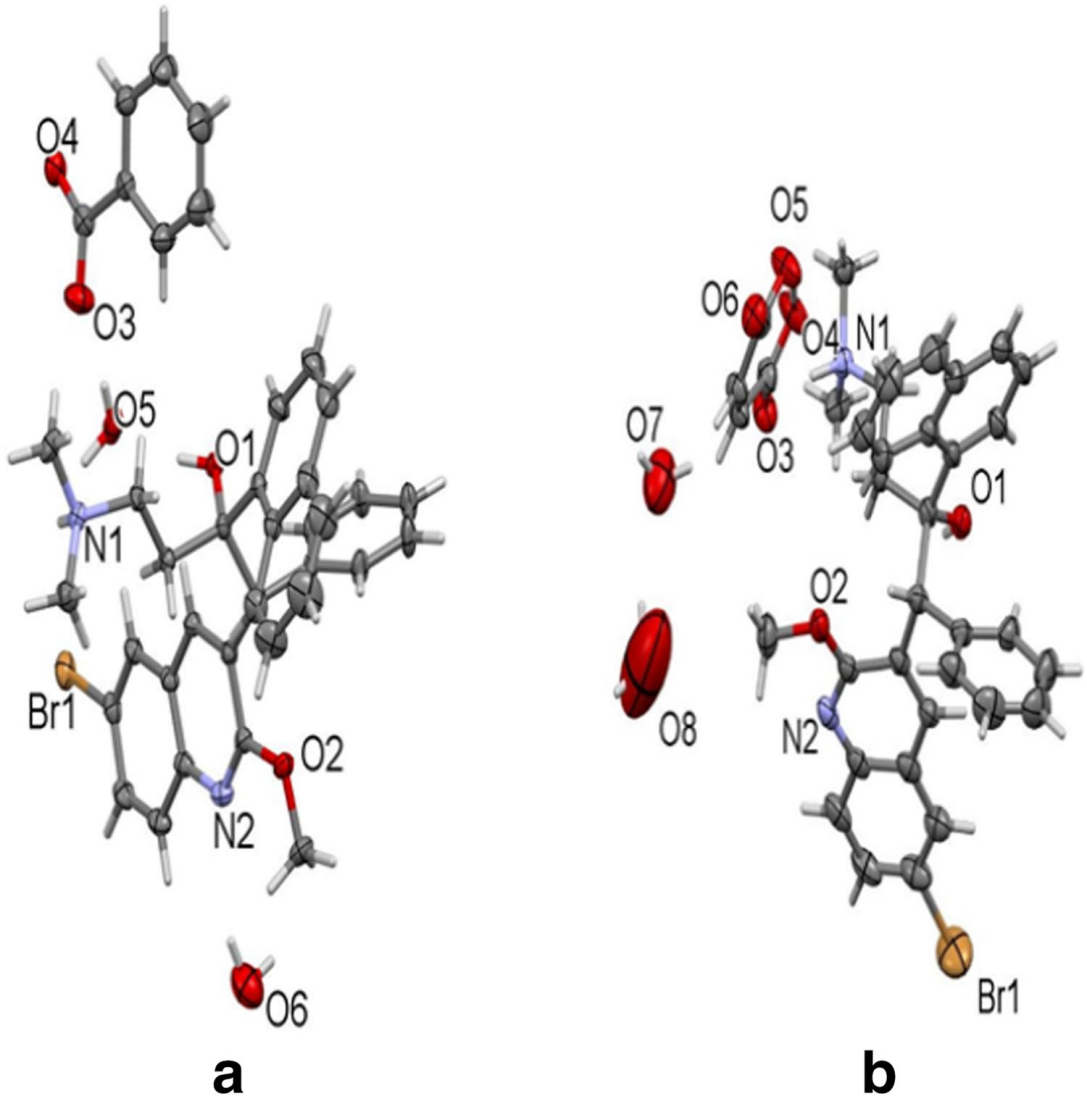
**a** Bedaquiline benzoate single crystal. One fully occupied and one partially occupied water molecule are present in the lattice. **b** Bedaquiline maleate as a 0.5 hydrate single crystal.

The corresponding single crystal structures for the benzoate and maleate salts are elucidated in Fig. 2. The single crystals for other solvates of benzoate and maleate (Fig. X), hydrochloride and besylate (Fig. XI) salts, are found in the ESM. No single crystals were solved for the mesylate salt and lactate salts, so there were no Rietveld refinements for them.

### Melting Point/Range Data

The melting point ranges for some of the salts are documented in Table IV below.

**Table IV:** Melting ranges obtained for different salts from study experiments

Salt experiment	Melting range (°C)
Bedaquiline benzoate (BABQ) from acetone	128 ± 1
Bedaquiline benzoate (BABQ) from IPA water	134 ± 1
Bedaquiline base	174 ± 1
Benzoic acid	121 ± 1
Bedaquiline fumarate	185 ± 1
BABQ from IPA polymorph screen (PS)	133 ± 1
BABQ from methanol PS	119 ± 1
BABQ from ethanol PS	128 ± 1
Bedaquiline hydrochloride salt from acetone slow and fast evaporation experiments	163 ± 1
Bedaquiline hydrochloride salt from IPA slow evaporation experiment	163 ± 1
Bedaquiline maleate from THF	143 ± 1
BABQ from propyl alcohol (PA) slurry PS	127 ± 1
BABQ from methanol slurry PS	131 ± 1
BABQ from acetone/hexane as antisolvent PS	134 ± 1
BABQ from acetone/water as antisolvent PS	116 ± 1
BABQ from PA/water as antisolvent PS	128 ± 1
BABQ from DMF heated and cooled PS	126 ± 1

### DSC Data

**Fig. 3.**
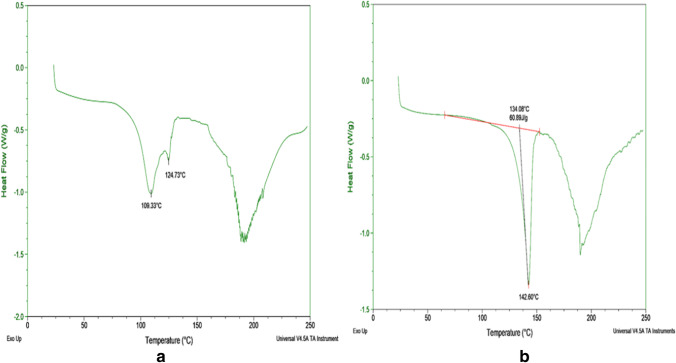
**a** Endothermic maxima at 109.33 °C and 124.73 °C corresponding to solvent evaporation and melt of benzoate salt prepared from acetonitrile. **b** Melting endotherm maximum from bedaquiline maleate salt at142.6 °C prepared from slow evaporation from THF.

The thermograms in Fig. 3 were generated from the benzoate and maleate salts DSC analysis. The thermograms for benzoate from 2-propanol, mesylate and besylate salts are found in the ESM (Fig. XII). The DSC results for the salts are close to the data obtained from the melting points determination.

### TGA Data

**Fig. 4.**
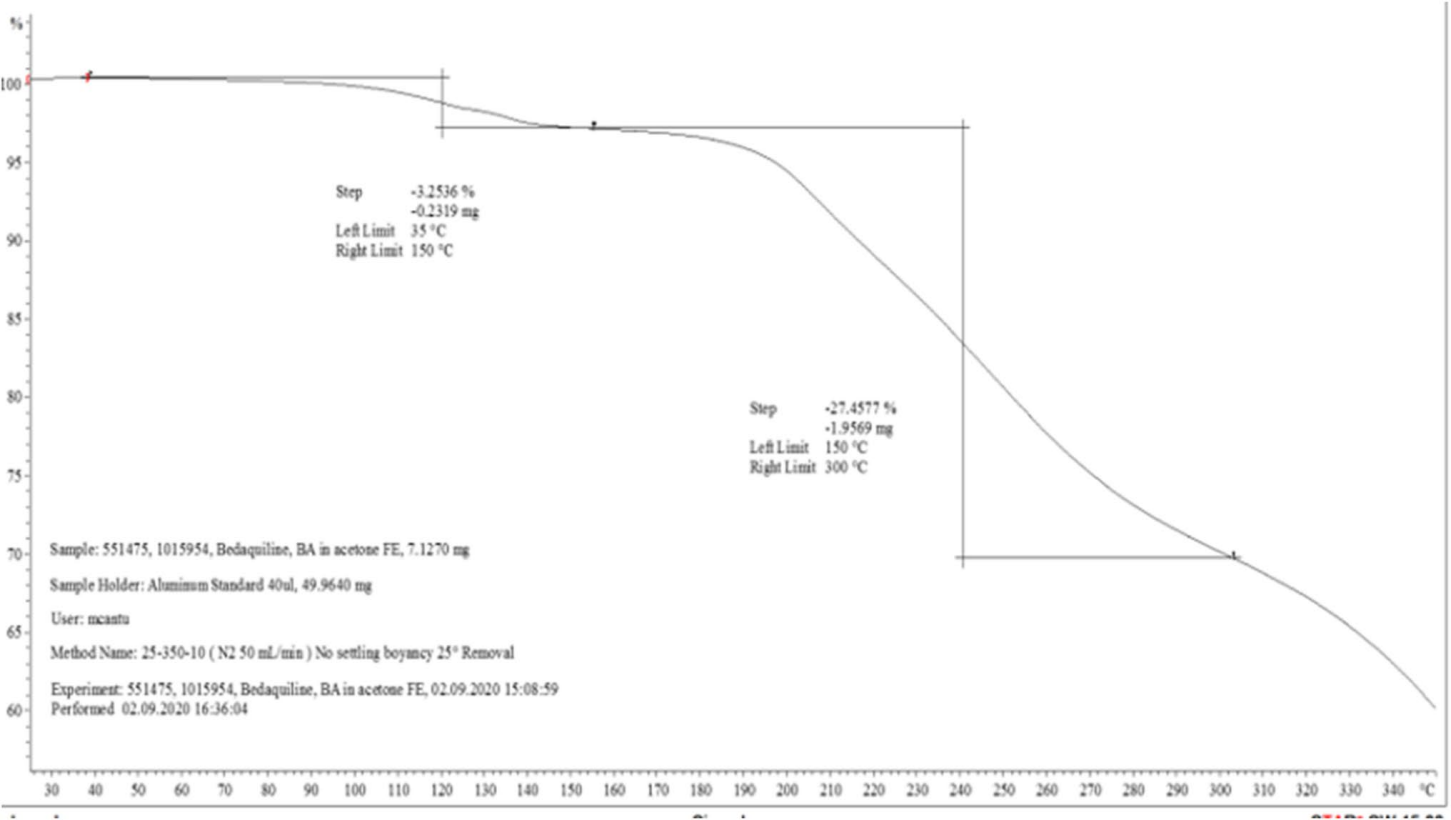
TGA data from benzoate crystalline salt with 1.17% hydrate. Percent weight loss was determined at 45 °C and 150 °C, respectively

During a desolvation phase, possible loss of adsorbed or bound solvents decreases the initial weights of samples. The initial weight loss for the benzoate salts, − 0.2319 mg (hydrate) and − 0.1247 mg (solvate), were attributable to loss of volatile components at the desolvation stages of the TGA. The percent weight loss determined for benzoate hydrate salt at 45 °C and 150 °C, respectively, is shown in Fig. 4. The TGA result for benzoate solvate is found in Fig. XIII (ESM).

### Hygroscopicity

The benzoate and maleate salts were non-hygroscopic (< 0.1% weight gain) upon exposure to 75% RH at ambient conditions for 25 days. Table XXII in the ESM section, provides details of water sorption results obtained for the new bedaquiline salts.

#### NMR

The stoichiometry of bedaquiline benzoate and fumarate salts, as well the numbers of protons are provided along with the chemical shifts of each peak in an earlier communication (12).

### HSOM Data

**Fig. 5.**
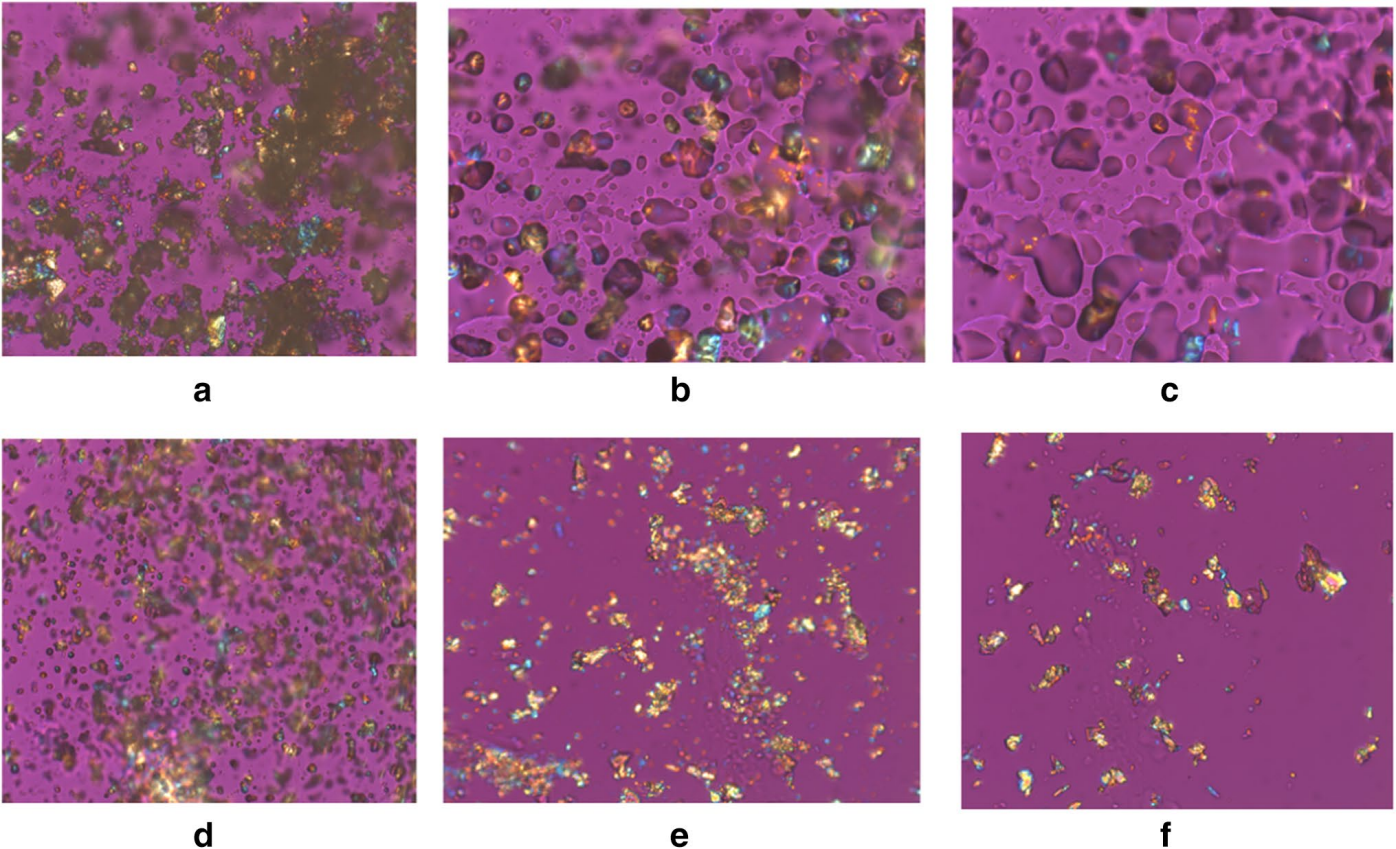
**a** HSOM data from a bedaquiline maleate crystal run (temperature: 32.3 °C, start of heating). **b** HSOM data from a bedaquiline maleate crystal run 1 (temperature: 130.6 °C, melting). **c** HSOM data from a bedaquiline maleate crystal run 1 (temperature: 131.5 °C, melting). **d** HSOM data from a bedaquiline maleate crystal run 2 (temperature: 124.7 °C, melting). **e** HSOM data from a bedaquiline maleate crystal run 3 (temperature: 36.2 °C, start of heating). **f** HSOM data from a bedaquiline maleate crystal run 3 (temperature: 119.9 °C, onset melting).

The results for the maleate salts experiments are summarized in Fig. 5 and Table V (ESM). Bedaquiline benzenesulfonate and methane sulfonate salts were also evaluated, and results are found in Fig. XIV (ESM).

### Water Content by KF

The benzoate salt formed from 2-propanol with water as the antisolvent, contained 3.37% moisture, while the 1.17 hydrated form contained 3.33% water.

### IR Spectroscopy

**Fig. 6.**
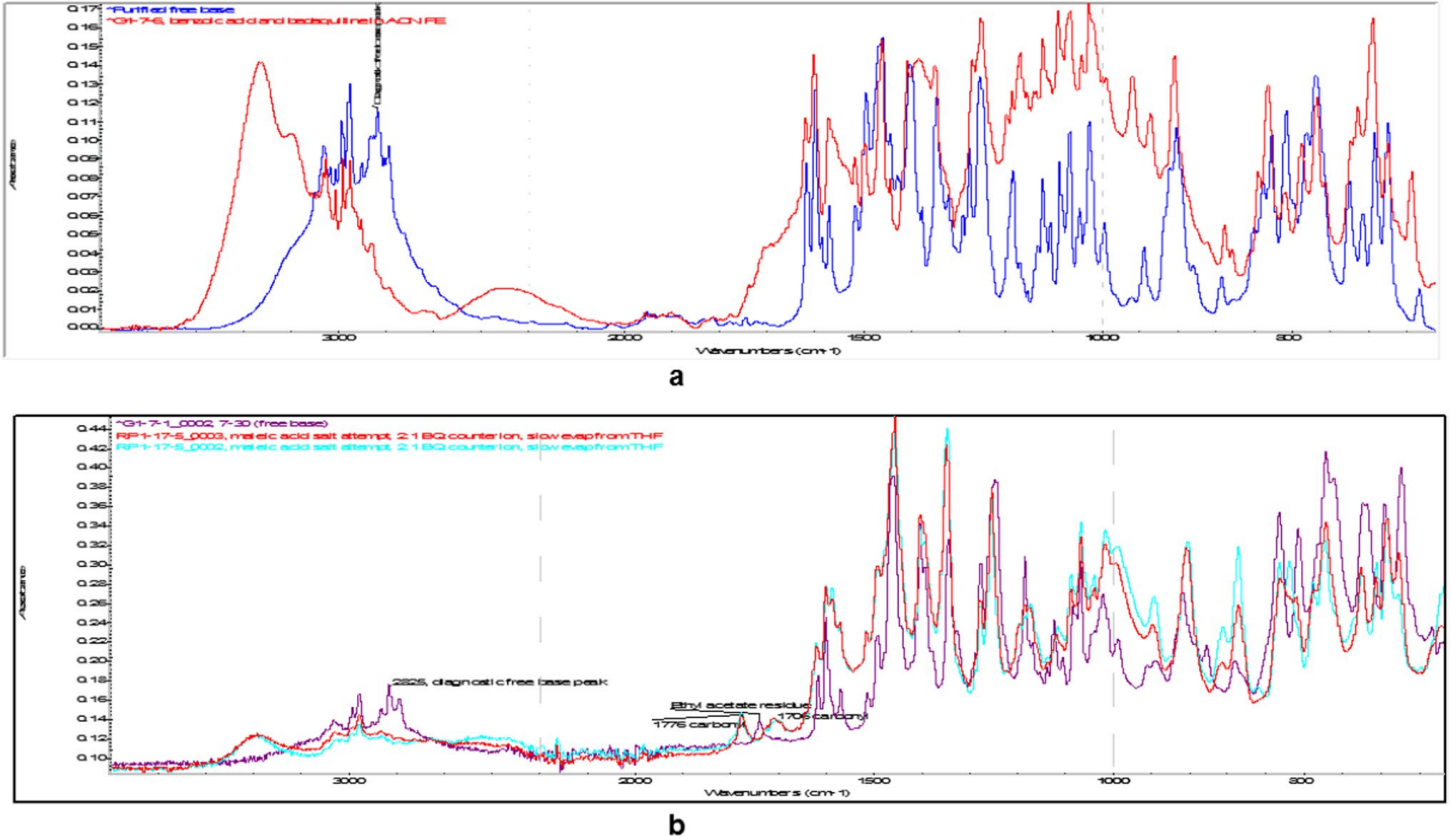
**a** Infrared spectra of bedaquiline benzoate and free base. **b** Infrared spectra of bedaquiline maleate and free base.

Successful salt formation was confirmed by IR microspectroscopy for the benzoate, maleate (Fig. 6), besylate, and mesylate bedaquiline salts (Fig. XV, ESM). Both ATR and reflection/absorption spectra were collected of the salts. The best quality spectrum for each salt is displayed in the figures below. No spectra were obtained for the HCl and lactate salts.

In each figure, the spectrum of the salt is compared with that of the free base. Salt formation is indicated by the loss of the peak at 2825 cm^−1^.

### Polymorph Screen Data

The following experimental conditions were used for the polymorph screen: evaporation, precipitation, crystallization, slurring, and melt crystallization attempts.

Some representative data from polymorph screen diffractograms are shown in the ESM for both benzoate (Figs. XVI, XVII, and XVIII) and maleate salts (Fig. XIX). None of the results revealed a new crystal form. PXRD from powdered samples displayed peaks that matched either the single crystals of the parent salt, the free base, or a mixture of both. These results suggest that no new polymorphs were formed. Tables XIX, XX, and X1 (ESM) are representative summaries of the results from the polymorph screens.

## DISCUSSIONS

Bedaquiline is used in the treatment of multi-drug resistant tuberculosis (TB) in adults (38). The commercially available salt is the fumarate marketed under the trade name Sirturo. The current study conducted a screen to discover other salts of bedaquiline that can be further developed in pharmaceutical manufacturing to make the product more available for TB treatment. A salt can be formed from acid–base reactions, and in the current study, potential salts of bedaquiline were investigated using the free base reactions with ten acids (salt formers). The bedaquiline molecule has two basic nitrogen atoms that can be protonated, the tertiary amine attached to the ethylene group, and the quinoline nitrogen atom. The two sites exhibit different basicity, so that protonation will most likely occur in the more basic amine site. Therefore, mono and dicationic salts of bedaquiline can be formed using acids with various strengths (12).

### Single Crystal Structures of the Salts Generated from the Screening Experiments

Following the salt screen experiments, crystalline salts of bedaquiline: benzoate, maleate, mesylate, besylate, and the hydrochloride, were isolated and their structures were determined using single crystal X-ray diffraction. The phase purity of the obtained bulk powder samples was investigated by PXRD. The weights of acids used corresponded to a 1:1 ratio equivalent to 30 mg (0.054 mmol) of bedaquiline base. Further experiments used a 1:2 ratio for succinic, maleic and malic acids.

Benzoate salts were recovered from experiments using acetone, acetonitrile, 2-propanol and methanol under slow and antisolvent (water and hexane) conditions. The three isolated benzoate salts were solvates, which though similar to each other, were isomorphous (the methanol and acetonitrile solvates were solved by isomorphous replacement from the hydrate). A comprehensive description of the hydrate and acetonitrile solvates has been published previously (12). Similarly, two single crystal structures were determined for the maleate salt; a THF solvate and a 0.5 hydrate form. Similarly, crystal structures could also be determined for the besylate and hydrochloride salts. To explore the possibility to protonate the two amine sites on the bedaquiline molecule, 2 and 3 equivalents of a strong acid, HCl were added to 1 equivalent of the base in acetone. The PXRD patterns of the salts formed matched the pattern from the single crystal structure for the hydrochloride form, with only the tertiary amine protonated.

### X-ray Diffractions from Bedaquiline Salts

In solid-state chemistry, X-ray crystallography can be used to elucidate the structure of solids. This illustrates the existing crystalline packings and provides images of solids with their atomic dimensions using computerized Fourier analysis. Crystalline powders, unlike amorphous materials, exhibit ordered structures; this enables them to display distinct diffraction patterns or characteristic fingerprints. The location of atoms relative to their unit cells can be visualized through the structure of their single crystals. Description of single-cell structures also measures intensities of the diffracted beams from the various crystal planes. The diffraction Intensity is a measure of the electron density of the atoms in the diffracting planes (13). The tables that fully describe the experimental details of the XRDs generated from the salt screens are in the ESM of this paper (Tables V1, VII, VIII, IX, X, XI, XII, and XIII). For the bedaquiline benzoate single crystal, three forms were generated. As examples:

The hydrated benzoate form had one fully occupied and one partially occupied water molecule in the lattice. Positions of the H atoms of the fully occupied H_2_O molecule were freely refined. Those of the partially occupied water molecule were initially refined and O–H and H…H distances were restrained to 0.84(2) and 1.36(2). Angstrom, respectively, while a damping factor was applied. The position of H6E was further restrained based on hydrogen bonding considerations. In the final refinement cycles the damping factor was removed and the H atoms were set to ride on the carrying oxygen atom. Subject to these conditions, the occupancy rate refined to 0.166(7), see Table VI (ESM).

The structure for the acetonitrile solvated form was solved by isomorphous replacement from its 1.17 hydrate analogue, which features one fully occupied and one partially occupied water molecule. In the present structure, the partially occupied water molecule was replaced by a partially occupied acetonitrile molecule. In the absence of the acetonitrile molecule the neighboring benzene ring of the benzoate anion tilts slightly to move towards the void left by the absent solvate molecule. The two disordered benzene moieties were restrained to have similar geometries. *U*_*ij*_ components of ADPs for disordered atoms closer to each other than 2.0 Angstrom were restrained to be similar. ADPs of the ipso carbon atoms, which occupy nearly identical positons, were constrained to be identical. Subject to these conditions the occupancy ratio refined to 0.742(7) to 0.258(7). Positions of the H atoms of the H_2_O molecule were freely refined, and O–H distances were restrained to 0.84(2) Angstrom, see Table VII (ESM).

Similarly, the structure for bedaquiline benzoate single crystal from methanol was solved by isomorphous replacement starting from its 1.17 hydrate analog. The partial (17%) occupied water molecule in the hydrate was replaced by a more prevalent methanol molecule. Occupancy refinement for the methanol molecule yielded 76.7(8)% occupancy, see Table VIII (ESM).

For bedaquiline maleate single crystal with 0.5 hydrate, the position of the hydromaleate acidic hydrogen atom was freely refined. Two partially occupied water molecules are situated in the asymmetric part of the unit cell. One in a general position, the other located on a twofold axis. They are hydrogen bonded to each other, and the one ion the general position is also H-bonded to O_3_ of the hydromaleate anion. Water H atom positions were refined and O–H and H…H distances were restrained to 0.84(2) and 1.36(2) Angstrom, respectively, and H atom positions were further restrained based on hydrogen bonding considerations. A damping factor was applied during refinement. In the final refinement cycles the damping factor was removed and the water H atoms were set to ride on their carrier oxygen atoms. Subject to these conditions the occupancy rates refined to 0.276(17) for O7 (general position) and 0.40(4) for O8 (twofold axis). Additional solvent accessible space is present in the crystal lattice (two times 123 cubic Angstrom, or 6.9% of the unit cell volume). No electron density was found inside the void space (A Platon Squeeze analysis corrected for 8 electrons in the combined void space), and the content of the void space was ignored, see Table XI (ESM).

Also, the bedaquiline maleate single crystal with THF solvate was refined as a 2-component inversion twin. The position of the hydromaleate acidic hydrogen atom was freely refined. The structure was solved from its 0.5 hydrate analogue by isomorphous replacement. Two THF molecules were refined as disordered. One in a 1:1 ratio around a twofold axis, the other in a general position. The three disordered moieties were restrained to have similar geometries. *U*_*ij*_ components of ADPs for disordered atoms closer to each other than 2.0 Angstrom were restrained to be similar. Subject to these conditions the occupancy ratio for the molecule in the general position refined to 0.587(16) to 0.413(16). See Table XII (ESM).

### Thermal Analysis and Water Determination

The DSC endothermic phase transition observed gave values that were comparable to the melting point ranges of the individual salts. There is a close relationship between DSC melt endotherms and melting points of organic materials. This association forms the basis for using some organic materials as standards in calibrating a DSC equipment (39). The thermograph endotherm for the benzoate salt showed to melt at 124.73 °C, and the range obtained with the melting point apparatus was 128 ± 1 °C. A close match was also observed for thermograph endotherm maleate melt of 143.02, and a melting range of 143 ± 1 using the Thomas Hoover Capillary apparatus. In addition, the hot-stage optical microscopy (HSOM) results supported the thermal observations for all salts.

TG was used to investigate if the materials exhibited weight changes and to detect weight changes due to events such as decomposition, sublimation, desolvation, and other chemical reactions (40). From the TGA analysis of the benzoate salt, some weight loss in the sample, suggested that the salt was a solvate or a hydrate. From the TGA results, the benzoate crystalline salt with 1.17% hydrate weight loss was − 0.2319 mg (− 3.2536%), while the acetonitrile form is unstable outside of solvent and converts to the mono-hydrate. Weight loss assuming a mono-hydrate (rather than 1.17 hydrate) gives 3.16. Theoretical calculation for weight loss for 1.17% water occupancy was generated for three scenarios. First assumption, if the benzoate salt had 1.17% water lost, the corresponding weight loss should be ~ 3.7%. For an alternate assumption, with 1% water lost, the corresponding weight loss should be ~ 3.13%. And for a final scenario, if the benzoate salt is 1% hydrated, with loss of 1%, then, the corresponding weight loss should be ~ 3.14%. These results were consistent with the description of the two single crystals isolated for benzoate, an acetonitrile solvate and a hydrated form (12).

There are two types of moisture associated with pharmaceutical solids. The sorbed (unbound) moisture are freely associated with the solids’ surfaces in form of liquid films, in large pores or in voids that exists between particles. Secondly, moisture that is incorporated into the lattice, usually as hydrates, and occupy predictable locations within the crystal. Loss on drying (LOD), is used to assess unbound moisture, while Karl Fischer will evaluate both bound and unbound water (41). Pharmaceutical manufacturers must demonstrate that bound moisture is not negatively altered during manufacturing (1). KF using the coulometric method was used to evaluate the water content of the benzoate salts. This method is effective in determining small amounts of water (42). The water content obtained for the benzoate 1.17 hydrate salt (3.33%) correlates with the TGA results, where the weight loss was 0.2319 mg (3.2536%). In addition to these techniques, hygroscopicity can also be measured by measuring mass gain upon exposure to an environment with a specified relative humidity. For both maleate and benzoate salts, the materials did not exhibit weight gain and were classified as non-hygroscopic.

### Solid-State Chemistry and Polymorph Screen

The solid-state chemistry of the API and drug product is identified during drug development, and this information forms critical data required for regulatory submissions, CTD. At the early stages of pharmaceutical drug development, it is acceptable to conduct a quick polymorphic screen to identify the form used in the IND for phase 1 clinical trials. To ensure supply of a controlled drug form, an intermediate screen is often required during the second phase. Finally, comprehensive screening should be conducted using several crystallization solvents and experimental conditions. This screen elucidates all important forms that can impact product physicochemical attributes; which is necessary for filling New Drug Applications and obtaining patents (13).

The purpose of a polymorph screen is to discover as many crystal forms as possible and to select the most stable and manufacturable form. There are three milestones in a polymorph screen. The first is to discover as many forms as possible, followed by evaluating the stability order of the forms. The last step is to study the physicochemical attributes, which can help select a leading candidate for drug development (13). The two most promising salt candidates, benzoate and maleate, were selected for polymorph evaluation based upon ease of formation, scalability, hygroscopicity, and suitable solid-state properties. In the current study, crystallization, evaporation, slurrying, crystallization from the amorphous phase, and precipitation were employed in the polymorph screen. The solvents used were selected based on differences in dielectric constant, which is a measure of polarity (43). Identification and selection of the most stable crystal form is essential for scale-up activities, because of its impact on pharmaceutical product quality and characteristics.

Bedaquiline is poorly soluble in water, so it was used as the major antisolvent media, along with hexanes, in the precipitation experiments. The refinement for XRD of powdered samples where either of these antisolvents were used as antisolvent to precipitate salts from solutions, did not produce any new forms for either the benzoate or maleate salts. The diffractograms matched the patterns calculated from the corresponding single crystals of the parent salts. The salts were dissolved in varying quantities of the screening solvents and were subjected to fast and slow evaporation in scintillation vials. Examination of the powdered XRD did not yield any new forms of the salts. Slurring the identified salts in different solvents did not encourage the formation of additional forms either. Furthermore, the amorphous samples obtained from some experiments were heated in the hot air oven at temperatures below the salts’ melting point, to initiate possible crystallization. The amorphous benzoate salt upon exposure to ethyl acetate became crystalline after 24 h when heated at 60 °C. Further investigation of the XRD of the powdered sample matched the single crystal for the original form of the benzoate salt. However, similar crystallization treatment of the amorphous samples from both 2-propanol and hexane polymorph screen experiments were not successful. The salts disproportionated to mixtures of the free base and the benzoate salt.

Similar to the benzoate salt, the maleate salt screening attempts did not result in any new forms. All attempts to generate new polymorphs resulted in either crystalline material which refined to previously-identified structures (12) or amorphous material. In addition, three experiments from n-propanol, acetone, and ACN, using water as the antisolvent, resulted in disproportionation to mostly free base (> 87% for all cases) plus hydrated material. Finally, single crystals of bedaquiline maleate solvates were inadvertently grown during the polymorph screen attempts and the structures successfully solved; they were similar in structure to the previously identified solvates with infinite solvent channels and a high degree of disorder. HSOM of these single crystals showed that solvent loss was rapid and loss of solvate molecules was observable for the crystals. The melting behaviour was consistent with previously obtained thermal data, with no noticeable difference between the crystals. The comparable melts of these materials support the idea that the crystal structures of the maleate solvates are isomorphically related and that the presence of solvent is not required to maintain these structures.

The polymorph screen of the new bedaquiline salts generated powder X-ray diffractograms that did not suggest the existence of other polymorphs. The screen for the benzoate salt showed that the most predominant crystal form in the powders matched that of the 1.17 hydrate isolated as single crystals from acetone, can, and IPA solvent experiments. The Rietveld Refinement for the maleate polymorph screens, also suggested that the predominant form matched the 0.5 hydrate single crystals. Xu *et al*. described the crystal forms and methods of preparation, in the patent document for bedaquiline fumarate (44).

Phase transformations can occur when amorphous materials or metastable polymorphs are converted to more stable crystalline forms. PXRD can be used to monitor these solid-state reactions, where the converted material usually has a different diffraction pattern (13). Amorphous benzoate salt from three polymorph screen experiments converted to the crystalline form when oven heated at 60 °C for 24 h. The heated/cooled experiment for the benzoate polymorph screen using ethyl acetate, phase transformed from an amorphous material to the crystalline salt. However, 2-propanol and hexane experiments were partially disproportionated into mixtures of the free base and benzoate crystals. This phase transformation, however, was not the case for the maleate salt; unfortunately, no re-crystallization was observed during the thermal cycling experiments and only amorphous material resulted. Similar phase transformation were reported for amorphous calcium carbonate, which converted to crystalline polymorphs (45). Further formation of polymorphs can be investigated using melt crystallization. This methodology was employed in the research that discovered two new forms of piroxicam (46). There are ongoing experiments investigating other possible polymorphs from melt crystallization, and the results will be included in future publications of the current study.

The current report does not include solubility data. The DRUGBANK website reported a low water solubility of 0.000193 mg/ml for bedaquiline fumarate (47). For such poorly soluble compounds, the solid-state properties of the API can be used in optimizing formulations to enhance their apparent solubility. Such transformations can impact blood levels, important for bioavailability and therapeutic levels (48, 49). Future studies should evaluate the solubility of the newly discovered salts in relevant media.

## CONCLUSIONS

A salt screen successfully generated five salts of bedaquiline, namely, benzoate, maleate, hydrochloride, besylate, and mesylate. The salts were formed from equimolar combinations of bedaquiline base and the counterions from the corresponding acids. For structural confirmation, X-ray powder diffractograms for the salts were provided, together with the single crystal structures. Further confirmatory tests conducted on the new salts using polarized and hot stage microscopy, DSC, TG, IR, and NMR were referenced. A polymorph screen conducted on the salts suggested no presence of additional polymorphs at the current stage. However, the absence of additional forms needs to be further verified in future studies.

## Conflict of Interest

The authors declare no conflict of interest.

## Supplementary Information

Below is the link to the electronic supplementary material.

Supplementary file1 (DOCX 5467 KB)
