# Supplementary material for: Salts and Polymorph Screens for Bedaquiline
Source: AAPS PharmSciTech. 2021 Aug 25;22(7):228. doi: 10.1208/s12249-021-02106-7 (PMC8387259; doi:10.1208/s12249-021-02106-7)
Supplement: Supplementary file 1 — Supplementary file1 (DOCX 5467 KB) [file 12249_2021_2106_MOESM1_ESM.docx]

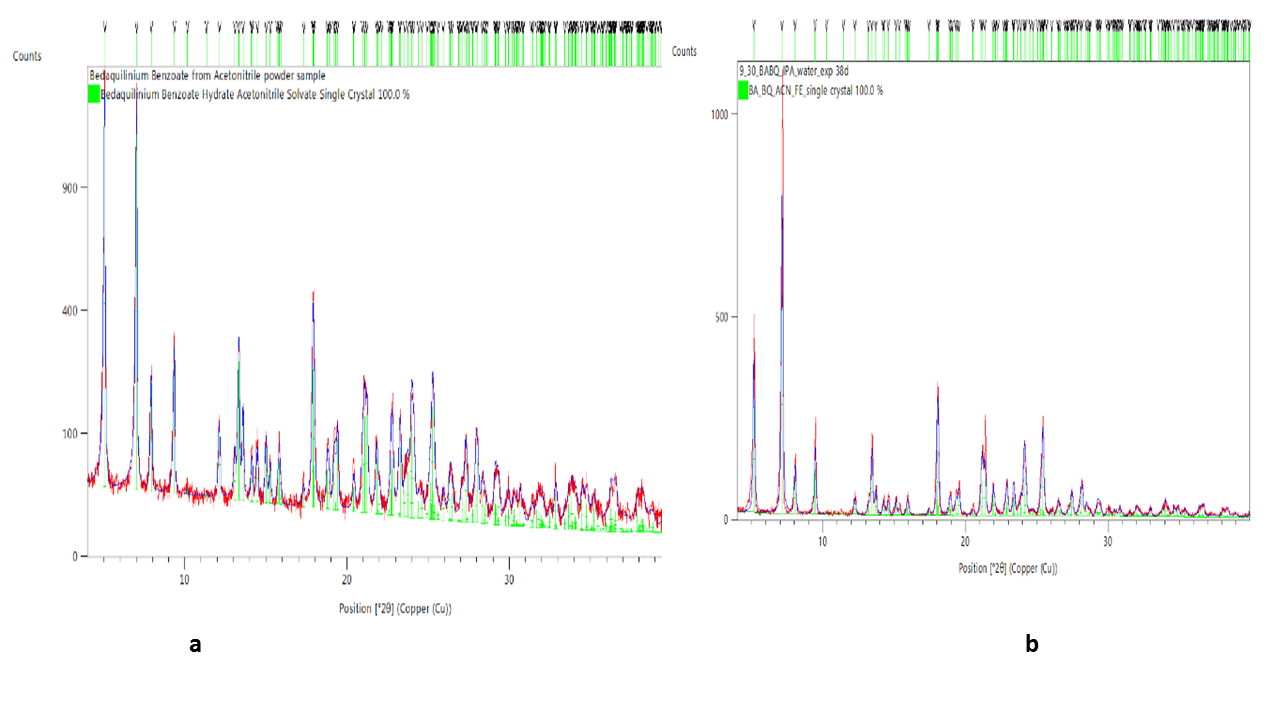
**Fig. VII (a-b)**

**a**: PXRD of benzoate salt from acetonitrile experiment gave a good fit when refined against the bedaquiline solvate single crystal. **b**: PXRD of benzoate salt from 2-Propanol_water antisolvent experiment gave a good fit when refined against the bedaquiline hydrate single crystal


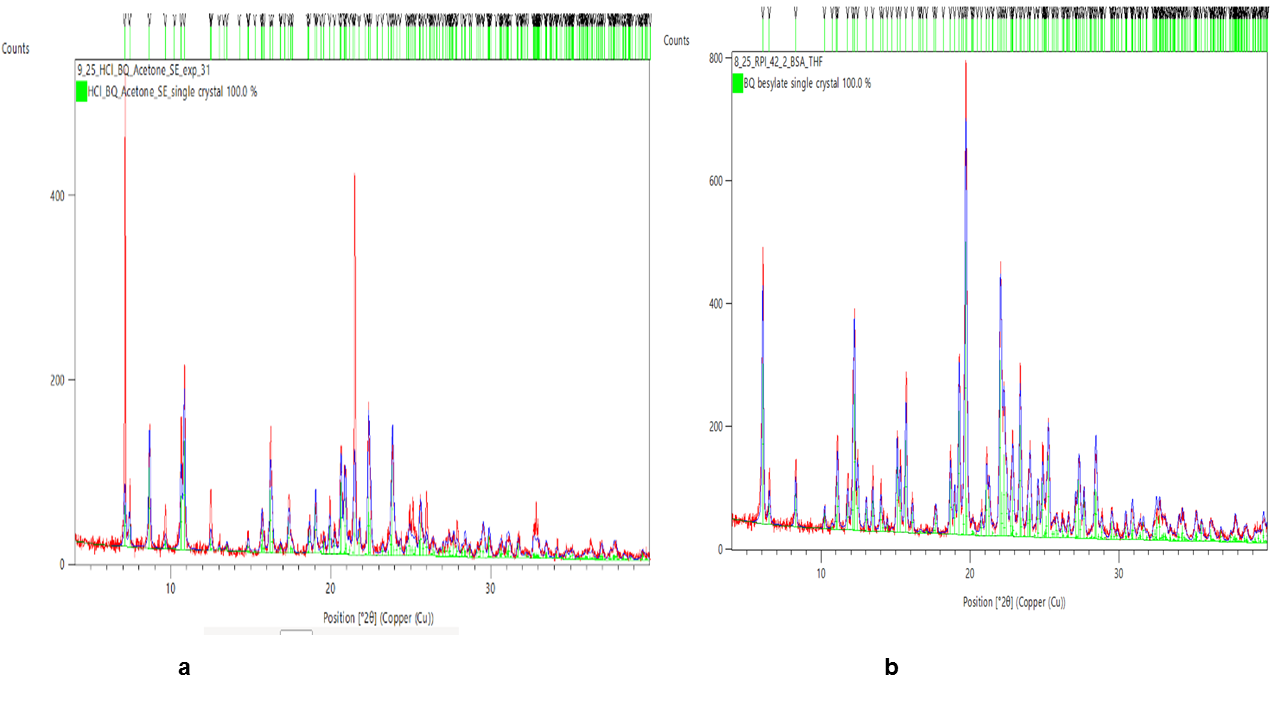


**Fig. VIII (a-b)**

**a**: PXRD of hydrochloride salt from acetone slow evaporation experiment gave a good fit when refined against the bedaquiline hydrochloride single crystal. **b**: PXRD of benzene sulfonic acid salt from THF experiment gave a good fit when refined against bedaquiline besylate single crystal.


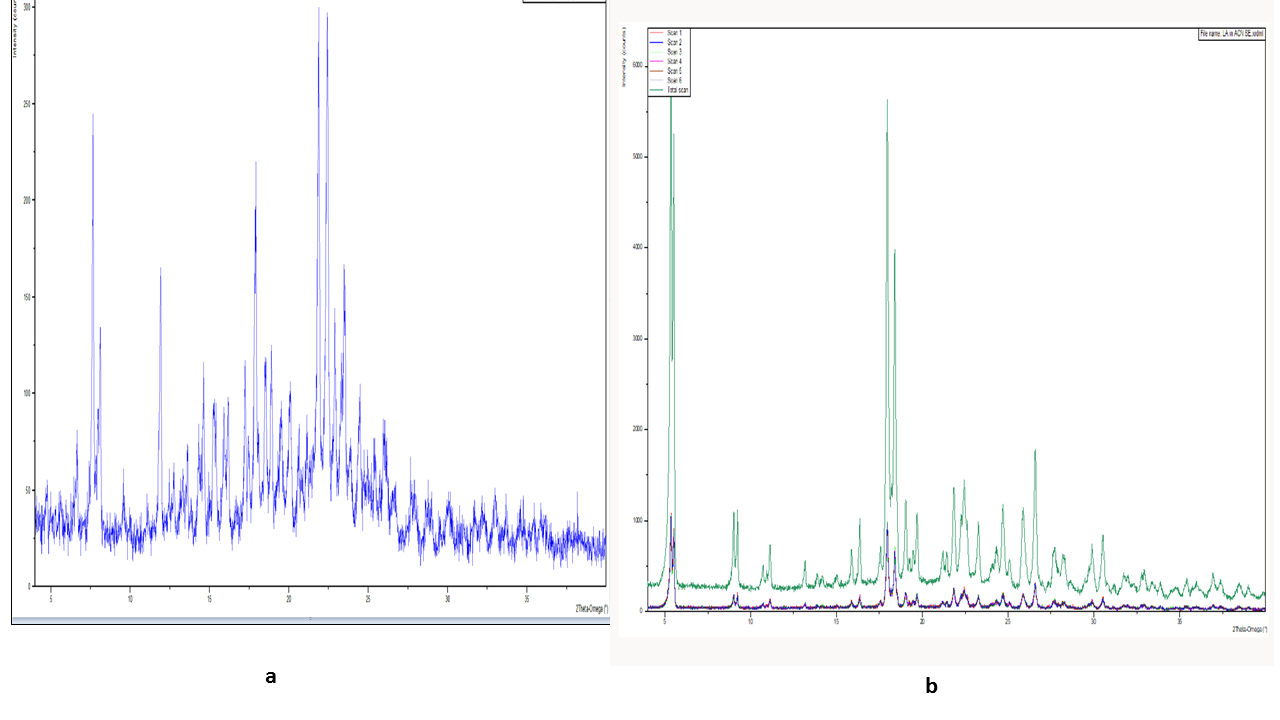


**Fig. IX (a-b)**

**a**: PXRD from methane sulfonic acid salt from THF experiment. **b**: PXRD from methane sulfonic acid salt from THF slow evaporation experiment


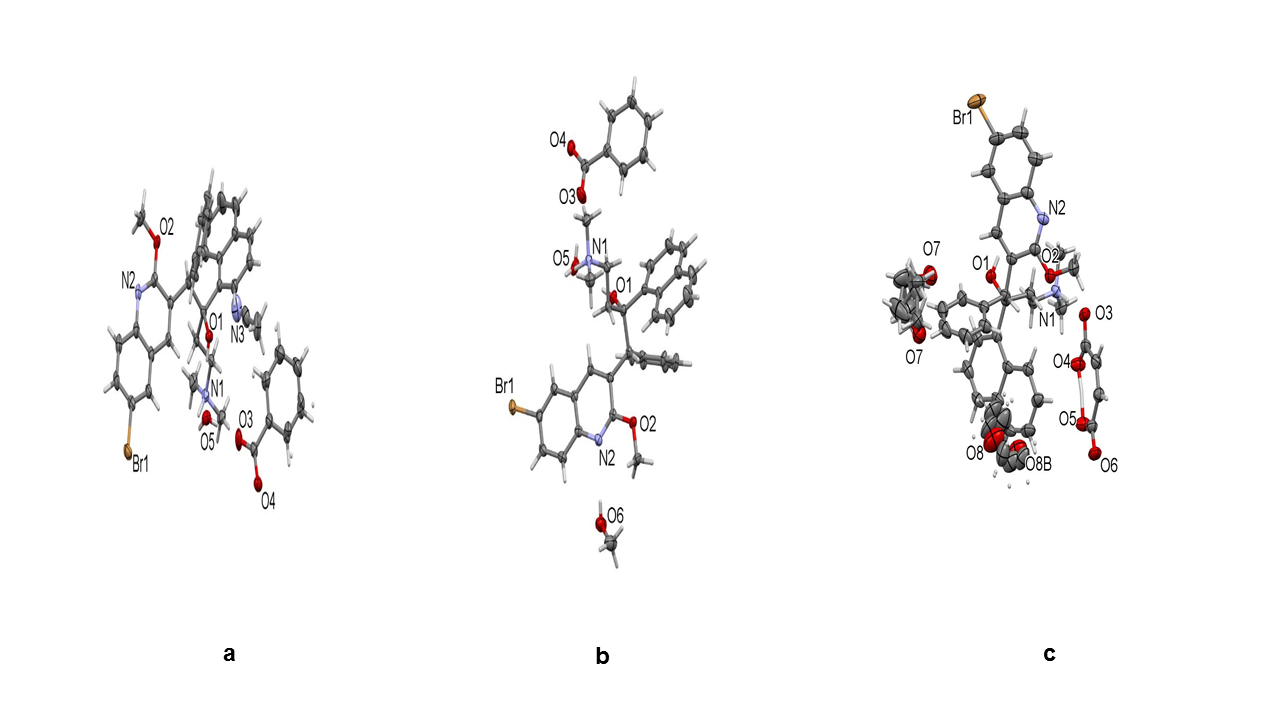


**Fig. X(a-c)**

**a**: bedaquiline benzoate acetonitrile solvate single crystal. **b**: bedaquiline benzoate single crystal from methanol. **c**: bedaquiline maleate (1:1) single crystal as a THF solvate containing 2 molecules of THF


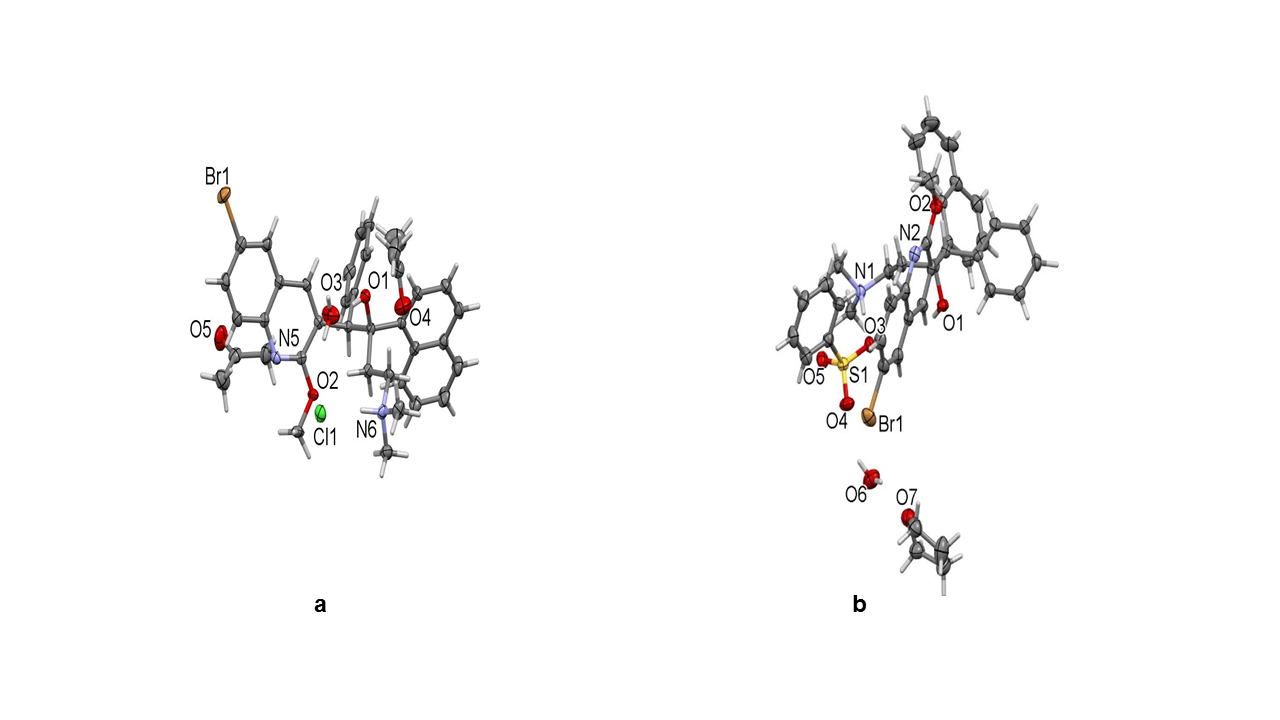


**Fig. XI (a-b)**

**a**: bedaquiline hydrochloride single crystal. **b**: bedaquiline besylate single crystal as a mixed solvate containing one mol of water plus one mol of THF.

**
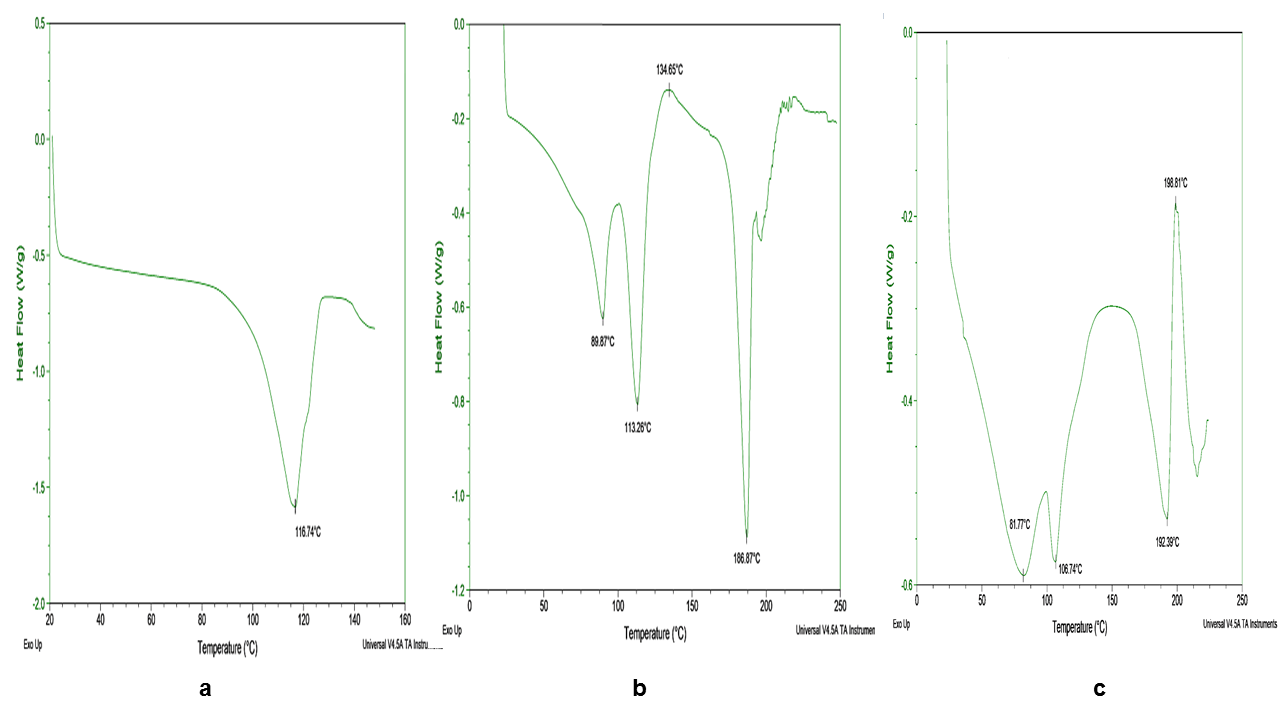
**

**Fig. XII (a-c)**

**a**: Endothermic maximum at 116.74 °C from the melt of bedaquiline benzoate crystal from 2-propanol using water as antisolvent experiment. **b**: Endothermic maxima at 89.87 °C, 113.26 °C, and 186.87 °C of Mesylate salt of bedaquiline obtained from slow evaporation from THF. **c**: Endothermic maxima at 81.77 °C, 106.74 °C, and 192.39 °C of Besylate salt of bedaquiline obtained from slow evaporation from THF


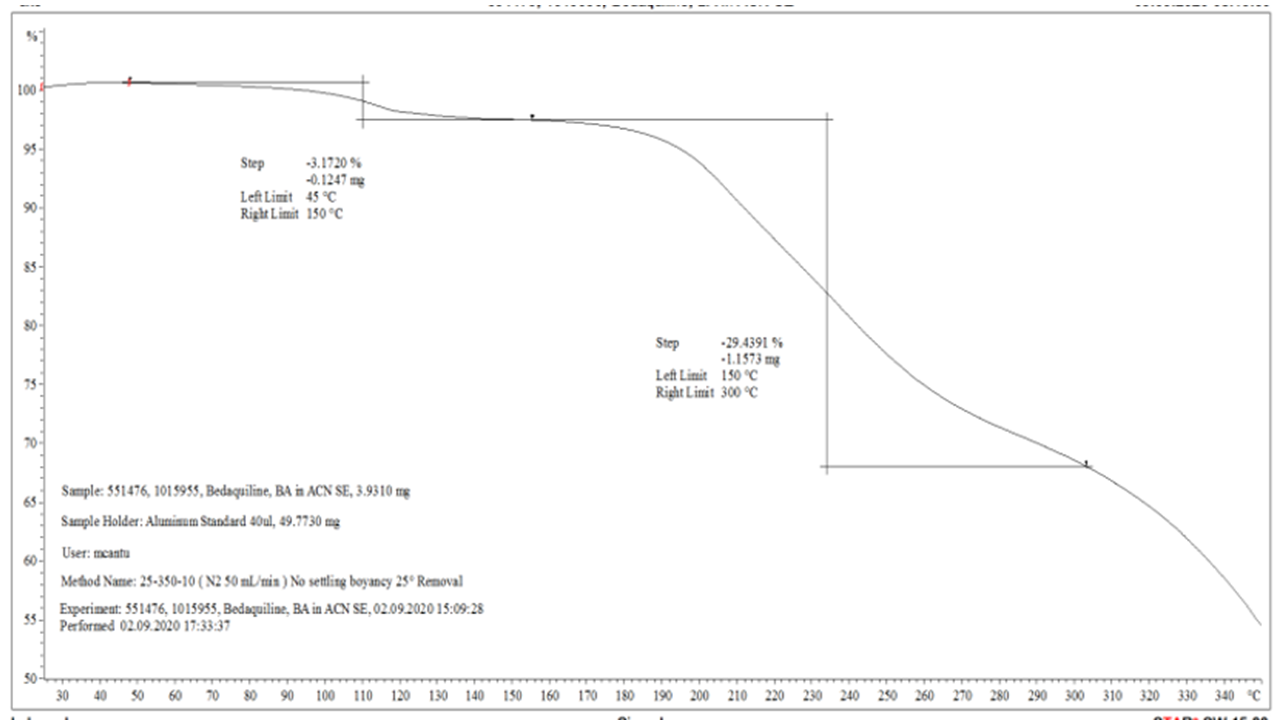


**Fig. XIII** TGA data from benzoate crystalline salt with acetonitrile solvate. Percent weight loss was determined at 45^o^C and 150^o^C respectively.

Table V: Sample and thermomicroscopy information.

| **Sample Description** | | | |
| --- | --- | --- | --- |
| Bedaquiline maleate (n-hexanes/acetone) | **Run 1** | **Run 2** | **Run 3 (in mineral oil** |
| **Images** | 1-91-2-001 to 1-91-2-005 | 1-91-2-006 to 1-91-2-011 | 1-91-2-012 to 1-91-2-016 |
|  |  |  |  |
| **Figures** | Figure 1 through Figure 5 | Figure 6 through Figure 11 | Figure 12 through Figure 16 |
|  |  |  |  |
| **Melting began** | 124.5 ˚C | 122.1 ˚C | 119.9 ˚C |
|  |  |  |  |
| **Melting complete** | 133.7 ˚C | 133.7 ˚C | 128.8 ˚C |
| Bedaquiline maleate (n-hexanes/ethyl acetate) | **Run 1** | **Run 2** | **Run 3 (in mineral oil)** |
| **Images** | 1-91-4-001 to 1-91-4-007 | 1-91-4-008 to 1-91-4-013 | 1-91-4-014 to 1-91-4-020 |
|  |  |  |  |
| **Figures** | Figure 17 through Figure 23 | Figure 24 through Figure 29 | Figure 30 through Figure 36 |
| **Melting began** | 122.1 ˚C | 122.8 ˚C | 118.4 ˚C |
|  |  |  |  |
| **Melting complete** | 133.6 ˚C | 133.6 ˚C | 128.3 ˚C |

**
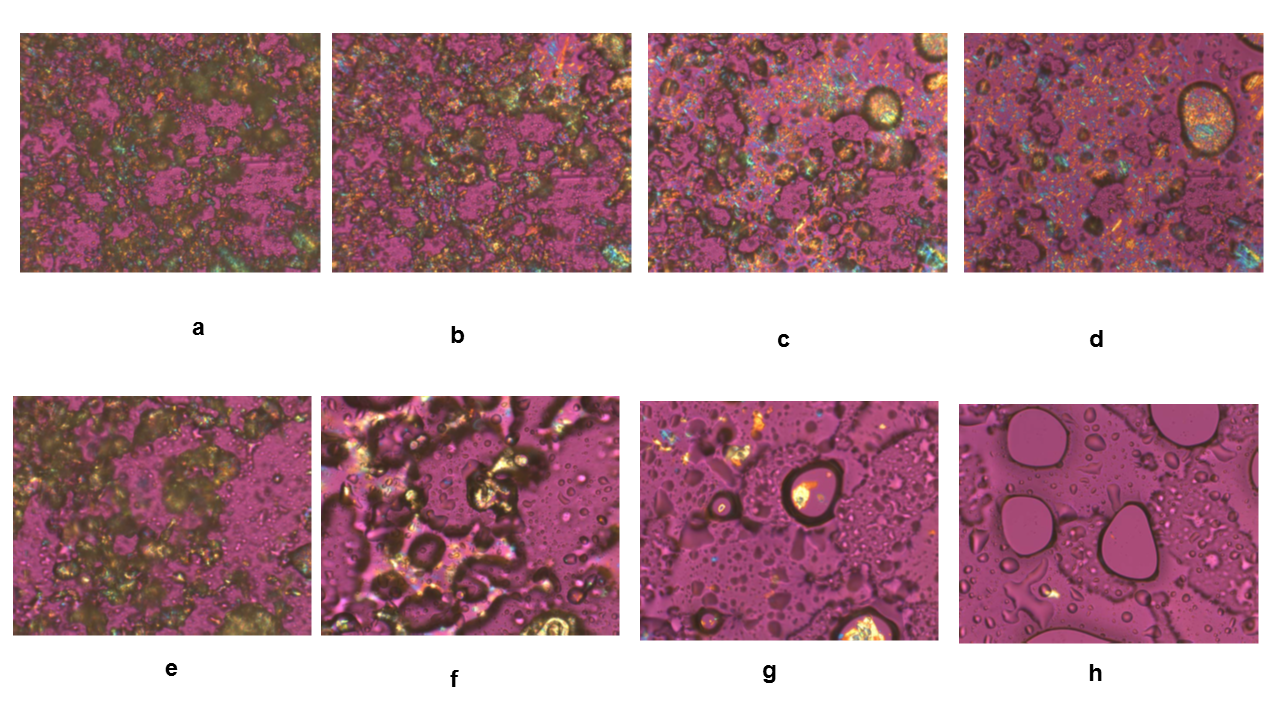
**

**Fig. XIV (a-h)**

HSOM images of bedaquiline benzenesulfonate using cross-polarized light with red compensator. **a:** 172.6 °C, wetting, melt onset. **b:** 180.1 °C, needles growing. **c**: 181.4 °C, melt/recrystallization. **d**: 182.5 °C, melt/recrystallization.

HSOM images of bedaquiline methane sulfonate using cross-polarized light with red compensator. **e**: 159.9 °C, changes in birefringence. **f**: 179.9 °C, start of melt. **g**: 180.2 °C, melting. h: 181.5 °C, melting.

**
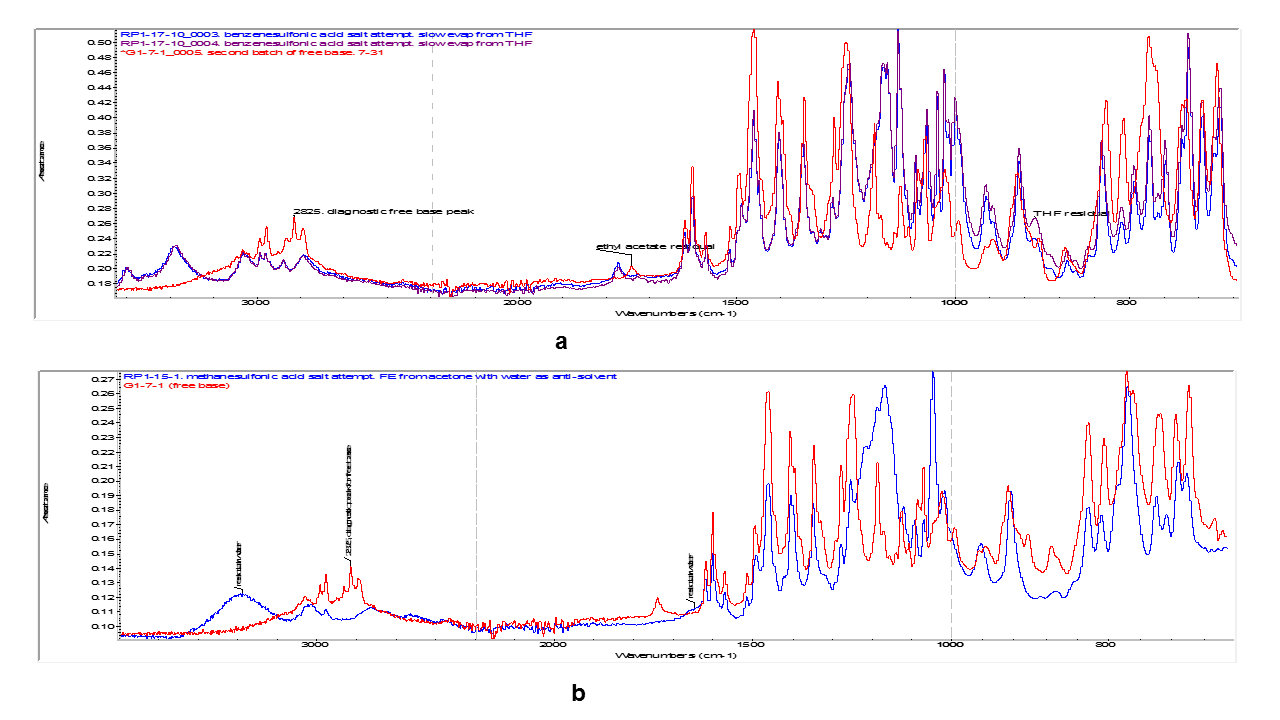
**

**Fig. XV (a-b)**

**a:** Infrared spectra of bedaquiline besylate and free base. **b**: Infrared spectra of bedaquiline mesylate and free base

**
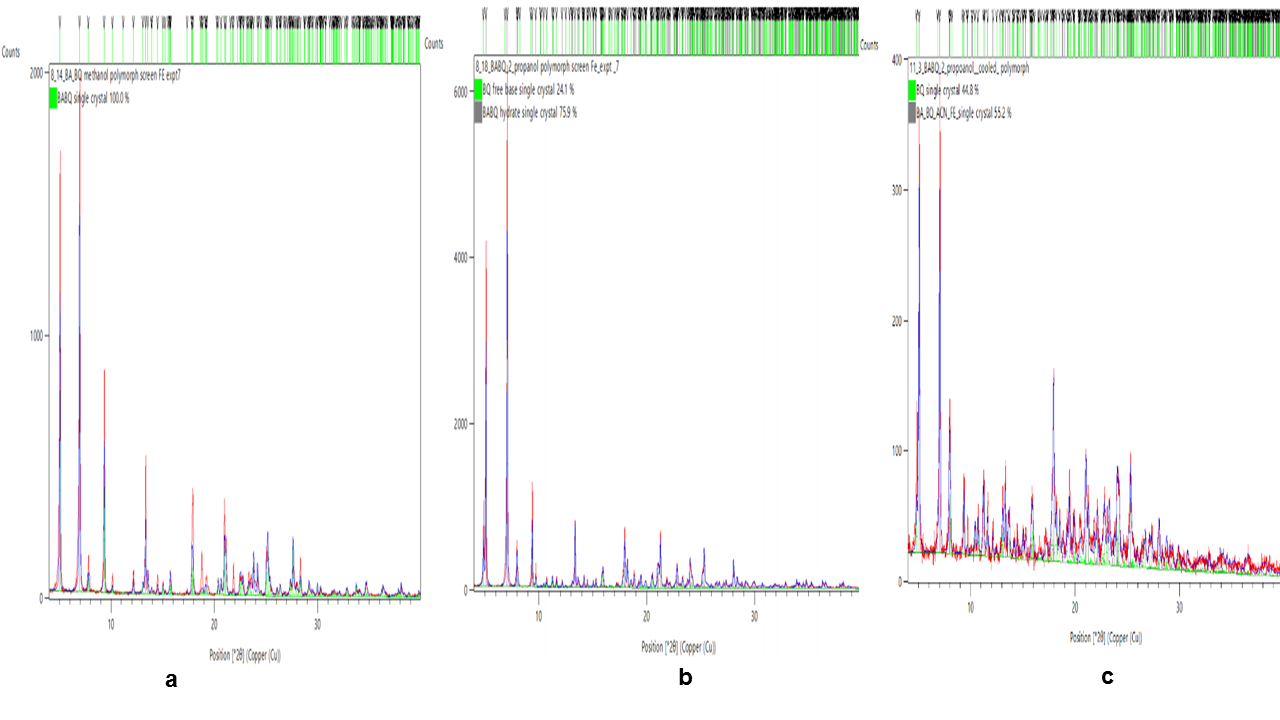
**

**Fig. XVI (a-c)**

**a**: benzoate salt polymorph screen in methanol fast evaporation experiment, gave similar peaks when refined against benzoate single crystal**. b**: Rietveld refinement of benzoate salt polymorph screen from 2-propanol fast evaporation experiment, gave peaks that suggested there were a mixture of benzoate and bedaquiline free base. **c**: Rietveld Refinement for polymorph screen for benzoate salt in 2-propanol, heated and cooled experimental condition. Shows mixture of benzoate salt and bedaquiline free base.


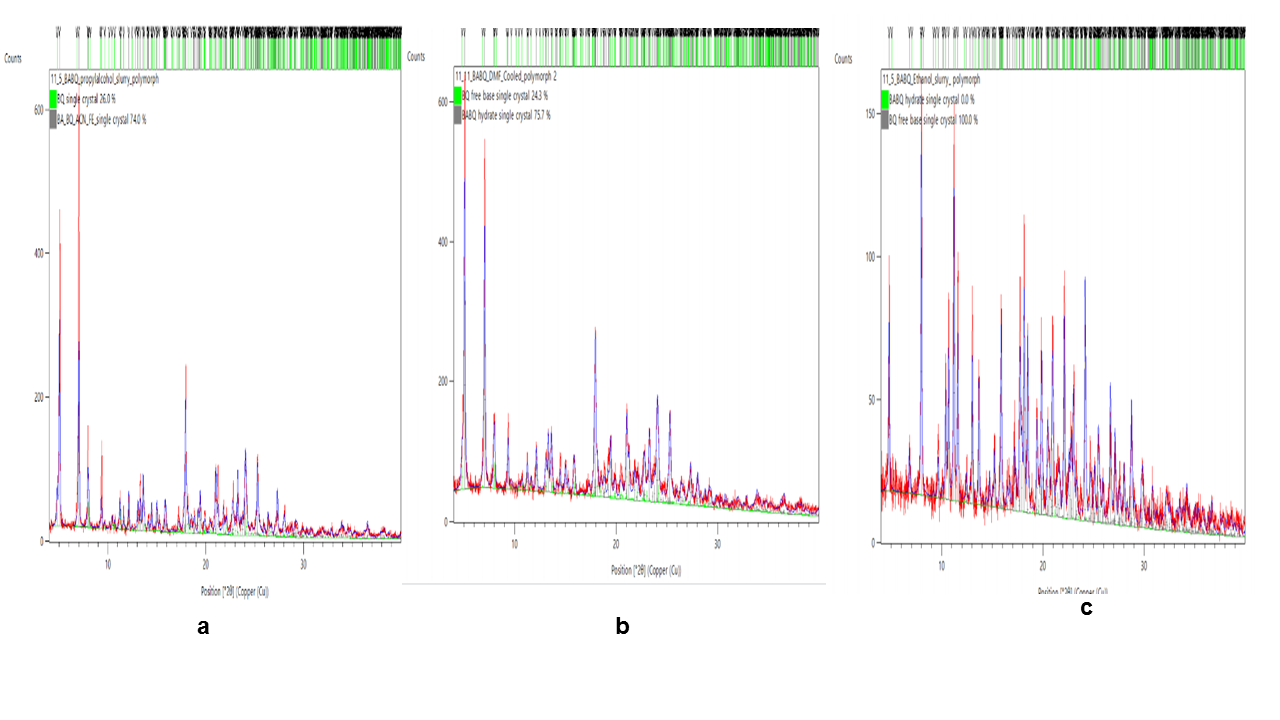


**Fig. XVII (a-c)**

**a**: Rietveld Refinement for polymorph screen for benzoate salt in propyl alcohol, 1-week slurry experimental condition. Shows mixture of benzoate salt and bedaquiline free base. **b**: Rietveld Refinement for polymorph screen for benzoate salt in DMF, heated and cooled experimental condition. Suggests a mixture of more benzoate and less free base. **c**: Rietveld Refinement for polymorph screen for benzoate salt in ethanol, I week slurry experimental condition. Salt disproportionated back to the free base.


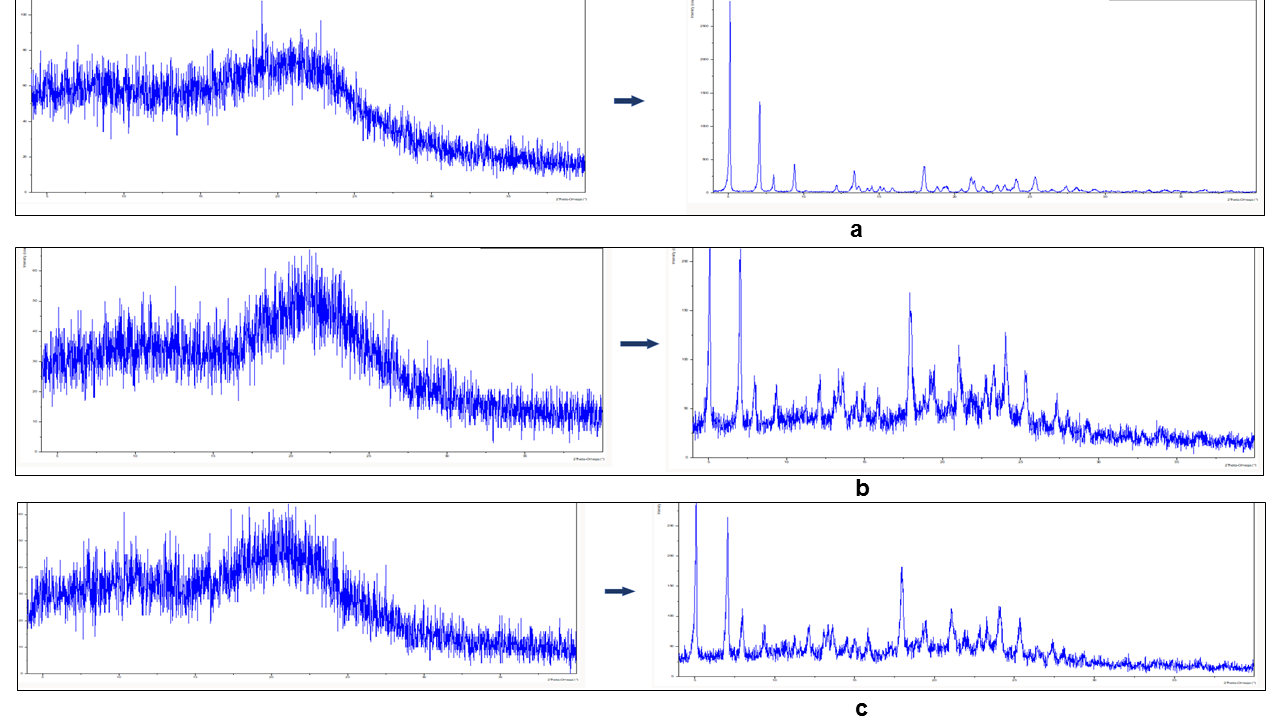


**Fig. XVIII (a-c)**

**a**: Amorphous benzoate (ethyl acetate heated/cooled polymorph) became crystalline after heating @ 60^o^C, 24hrs. **b**: Amorphous benzoate (propyl alcohol heated polymorph) became crystalline after heating @ 60^o^C, 24hrs. **c**: Amorphous benzoate (propyl alcohol heated polymorph) became crystalline after heating @ 60^o^C, 24hrs.


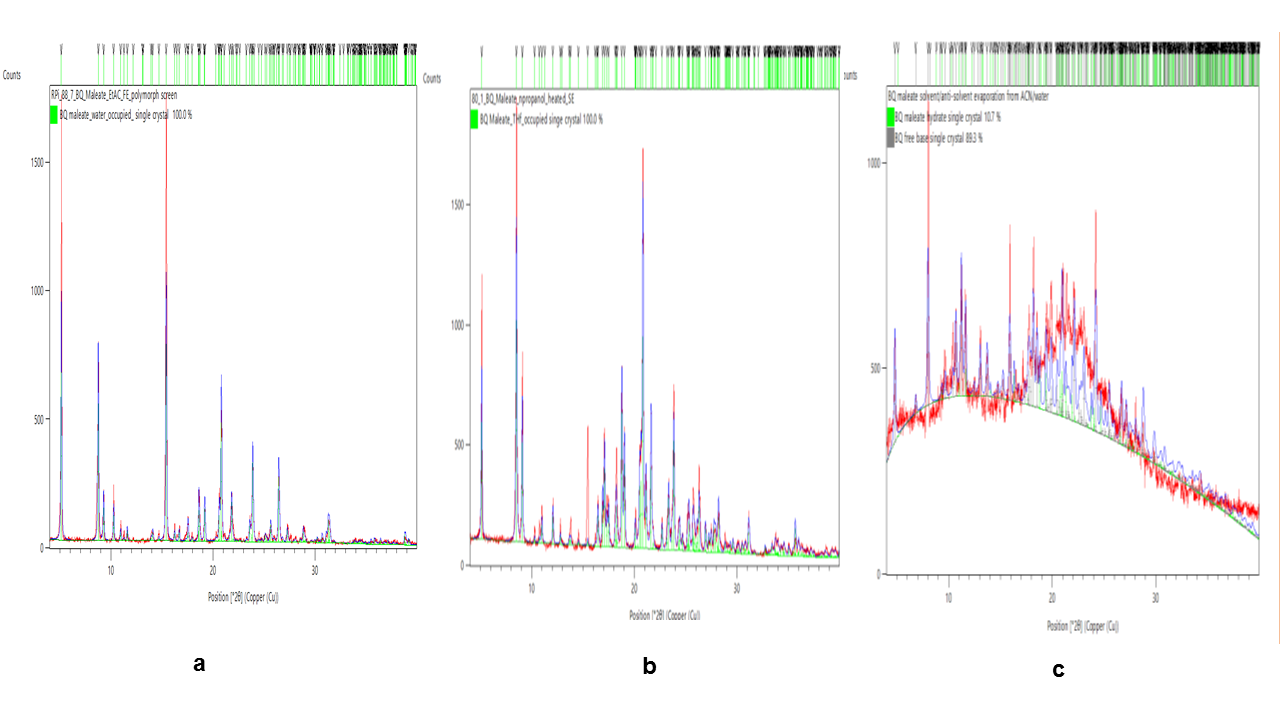


**Fig. XIX (a-c)**

**a**: PXRD of bedaquiline maleate from fast evaporation out of ethyl acetate, refined against the hydrate single crystal structure. **b**: PXRD of bedaquiline maleate from saturated solution/slow evaporation out of n-propanol, refined against the tetrahydofuran solvate single crystal structure. **c**: PXRD of bedaquiline maleate from solvent/anti-solvent experiment out of acetonitrile/water, refined against the free base and hydrate single crystal structures. A mixture of maleate salt and bedaquiline free base is apparent.


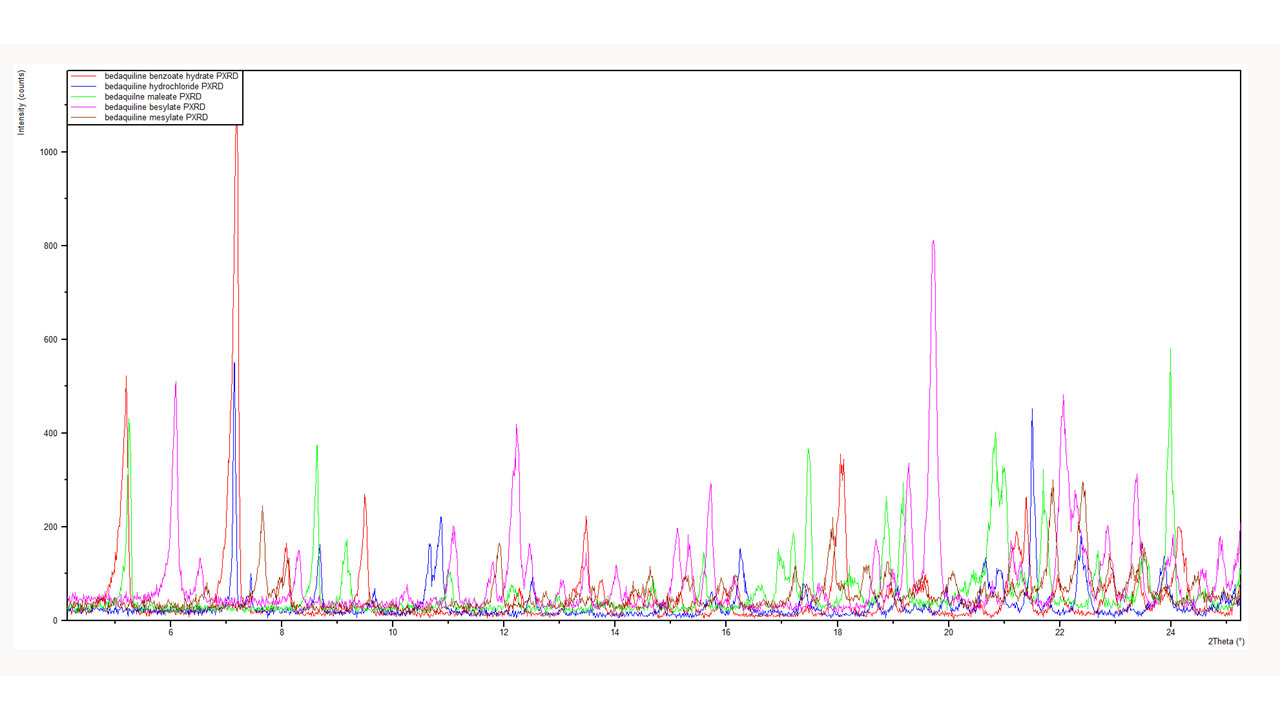


**Fig XX**

Overlay of PXRD for bedaquiline free base, hydrochloride, maleate, besylate, and mesylate salts. Salts diffracted x-ray and showing peaks intensities at identifiable 2-theta angles

**
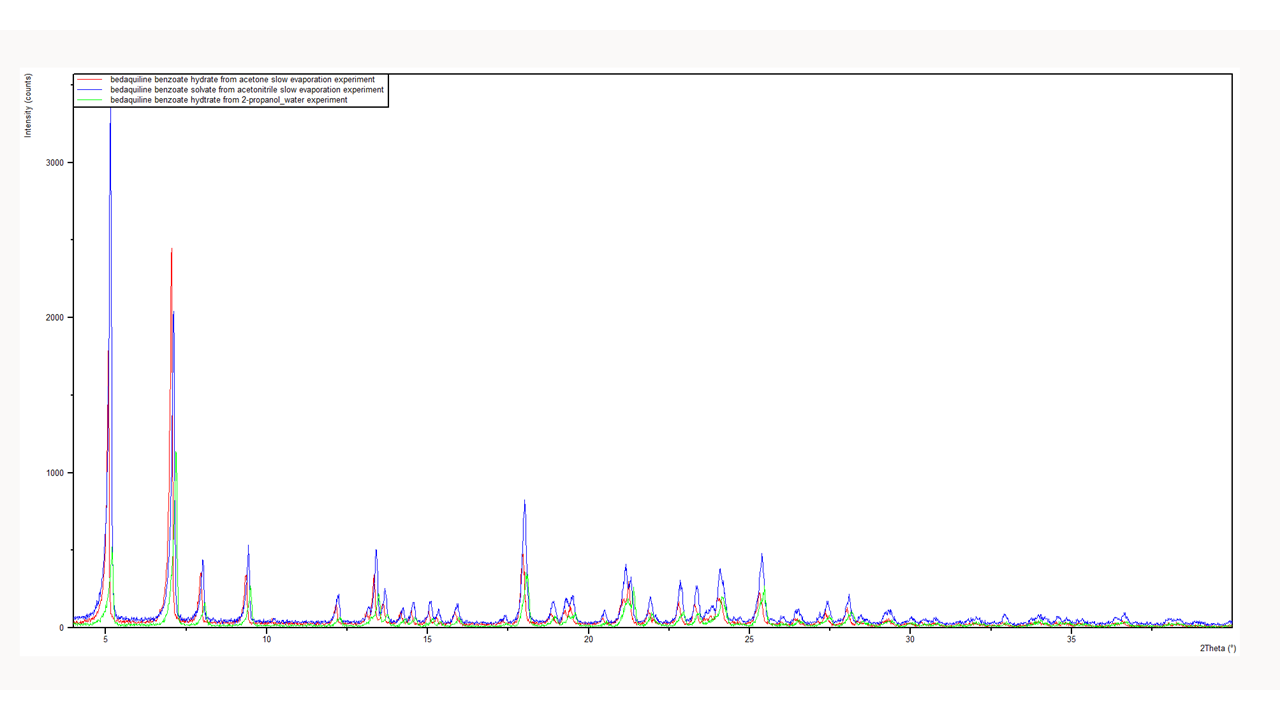
**

**Fig XXI**

Overlays of PXRD from benzoate hydrates and solvate salts from 3 routes of synthesis. Acetone slow evaporation, acetonitrile slow evaporation and 2-propano: water antisolvent experiments.

*Table VI: Experimental details for bedaquiline benzoate 1.17 hydrate single crystal*

|  | Bedaquiline benzoate 1.17 hydrate single crystal |
| --- | --- |
| Crystal data | |
| Chemical formula | C_32_H_32_BrN_2_O_2_·C_7_H_5_O_2_·1.166(H_2_O) |
| *M*_r_ | 698.70 |
| Crystal system, space group | Monoclinic, *P*2_1_ |
| Temperature (K) | 150 |
| *a*, *b*, *c* (Å) | 12.6384 (5), 7.9259 (3), 17.5249 (8) |
| β (°) | 99.8450 (17) |
| *V* (Å^3^) | 1729.63 (12) |
| *Z* | 2 |
| Radiation type | Mo *K*α |
| μ (mm^-1^) | 1.24 |
| Crystal size (mm) | 0.55 × 0.21 × 0.13 |
|  | |
| Data collection | |
| Diffractometer | Bruker AXS D8 Quest diffractometer with PhotonII charge-integrating pixel array detector (CPAD) |
| Absorption correction | Multi-scan, *SADABS* 2016/2: Krause, L., Herbst-Irmer, R., Sheldrick G.M. & Stalke D., J. Appl. Cryst. 48 (2015) 3-10 |
| *T*_min_, *T*_max_ | 0.638, 0.746 |
| No. of measured, independent and observed [*I* > 2σ(*I*)] reflections | 80228, 13080, 10456 |
| *R*_int_ | 0.049 |
| (sin θ/λ)_max_ (Å^-1^) | 0.770 |
|  | |
| Refinement | |
| *R*[*F*^2^ > 2σ(*F*^2^)], *wR*(*F*^2^), *S* | 0.032, 0.073, 1.02 |
| No. of reflections | 13080 |
| No. of parameters | 445 |
| No. of restraints | 5 |
| H-atom treatment | H atoms treated by a mixture of independent and constrained refinement |
| Δρ_max_, Δρ_min_ (e Å^-3^) | 0.36, -0.48 |
| Absolute structure | Flack x determined using 4051 quotients [(I+)-(I-)]/[(I+)+(I-)] (Parsons, Flack and Wagner, Acta Cryst. B69 (2013) 249-259). |
| Absolute structure parameter | 0.006 (3) |

Computer programs: Apex3 v2019.1-0 (Bruker, 2019), *SAINT* V8.40A (Bruker, 2019), SHELXM (Sheldrick, 2008), *SHELXL2018*/3 (Sheldrick, 2015, 2018), SHELXLE Rev1117 (Hübschle *et al.*, 2011).

Table VII: Experimental details for bedaquiline benzoate acetonitrile solvate single crystal

|  | Bedaquiline benzoate acetonitrile solvate single crystal |
| --- | --- |
| Crystal data | |
| Chemical formula | C_32_H_32_BrN_2_O_2_·C_7_H_5_O_2_·0.742(C_2_H_3_N)·H_2_O |
| *M*_r_ | 726.10 |
| Crystal system, space group | Monoclinic, *P*2_1_ |
| Temperature (K) | 150 |
| *a*, *b*, *c* (Å) | 12.8661 (8), 8.0386 (5), 17.4704 (10) |
| β (°) | 101.093 (3) |
| *V* (Å^3^) | 1773.13 (19) |
| *Z* | 2 |
| Radiation type | Cu *K*α |
| μ (mm^-1^) | 1.97 |
| Crystal size (mm) | 0.31 × 0.05 × 0.05 |
|  | |
| Data collection | |
| Diffractometer | Bruker AXS D8 Quest diffractometer with PhotonIII_C14 charge-integrating and photon counting pixel array detector |
| Absorption correction | Multi-scan, *SADABS* 2016/2: Krause, L., Herbst-Irmer, R., Sheldrick G.M. & Stalke D. (2015). J. Appl. Cryst. 48, 3-10. |
| *T*_min_, *T*_max_ | 0.599, 0.754 |
| No. of measured, independent and observed [*I* > 2σ(*I*)] reflections | 39739, 7360, 6750 |
| *R*_int_ | 0.060 |
| (sin θ/λ)_max_ (Å^-1^) | 0.639 |
|  | |
| Refinement | |
| *R*[*F*^2^ > 2σ(*F*^2^)], *wR*(*F*^2^), *S* | 0.035, 0.085, 1.06 |
| No. of reflections | 7360 |
| No. of parameters | 515 |
| No. of restraints | 195 |
| H-atom treatment | H atoms treated by a mixture of independent and constrained refinement |
| Δρ_max_, Δρ_min_ (e Å^-3^) | 0.40, -0.50 |
| Absolute structure | Flack x determined using 2778 quotients [(I+)-(I-)]/[(I+)+(I-)] (Parsons, Flack and Wagner, Acta Cryst. B69 (2013) 249-259). |
| Absolute structure parameter | 0.004 (8) |

Computer programs: Apex3 v2019.1-0 (Bruker, 2019), *SAINT* V8.40A (Bruker, 2019), *SHELXS97* (Sheldrick, 2008), *SHELXL2018*/3 (Sheldrick, 2015, 2018), SHELXLE Rev1117 (Hübschle *et al.*, 2011).

Table VIII: Experimental details of bedaquiline benzoate methanol solvate single crystal

|  | Bedaquiline benzoate methanol solvate single crystal |
| --- | --- |
| Crystal data | |
| Chemical formula | C_32_H_32_BrN_2_O_2_·C_7_H_5_O_2_·0.767(CH_4_O)·H_2_O |
| *M*_r_ | 720.20 |
| Crystal system, space group | Monoclinic, *P*2_1_ |
| Temperature (K) | 150 |
| *a*, *b*, *c* (Å) | 12.7037 (3), 7.9088 (2), 17.6589 (4) |
| β (°) | 100.9743 (12) |
| *V* (Å^3^) | 1741.76 (7) |
| *Z* | 2 |
| Radiation type | Cu *K*α |
| μ (mm^-1^) | 2.01 |
| Crystal size (mm) | 0.16 × 0.15 × 0.13 |
|  | |
| Data collection | |
| Diffractometer | Bruker AXS D8 Quest diffractometer with PhotonIII_C14 charge-integrating and photon counting pixel array detector |
| Absorption correction | Multi-scan, *SADABS* 2016/2: Krause, L., Herbst-Irmer, R., Sheldrick G.M. & Stalke D., J. Appl. Cryst. 48 (2015) 3-10 |
| *T*_min_, *T*_max_ | 0.653, 0.754 |
| No. of measured, independent and observed [*I* > 2σ(*I*)] reflections | 13551, 6718, 6217 |
| *R*_int_ | 0.037 |
| (sin θ/λ)_max_ (Å^-1^) | 0.639 |
|  | |
| Refinement | |
| *R*[*F*^2^ > 2σ(*F*^2^)], *wR*(*F*^2^), *S* | 0.034, 0.076, 1.04 |
| No. of reflections | 6718 |
| No. of parameters | 456 |
| No. of restraints | 1 |
| H-atom treatment | H atoms treated by a mixture of independent and constrained refinement |
| Δρ_max_, Δρ_min_ (e Å^-3^) | 0.39, -0.30 |
| Absolute structure | Flack x determined using 2387 quotients [(I+)-(I-)]/[(I+)+(I-)] (Parsons, Flack and Wagner, Acta Cryst. B69 (2013) 249-259). |
| Absolute structure parameter | 0.018 (9) |

Computer programs: Apex3 v2019.1-0 (Bruker, 2019), *SAINT* V8.40A (Bruker, 2019), *SHELXS97* (Sheldrick, 2008), *SHELXL2018*/3 (Sheldrick, 2015, 2018), SHELXLE Rev1183 (Hübschle *et al.*, 2011).

Table IX: Experimental details for bedaquiline hydrochloride single crystal

|  | Bedaquiline hydrochloride single crystal |
| --- | --- |
| Crystal data | |
| Chemical formula | C_32_H_32_BrN_2_O_2_·2(C_3_H_6_O)·Cl·H_2_O |
| *M*_r_ | 726.13 |
| Crystal system, space group | Orthorhombic, *P*2_1_2_1_2_1_ |
| Temperature (K) | 150 |
| *a*, *b*, *c* (Å) | 11.0424 (6), 13.4834 (6), 24.5720 (13) |
| *V* (Å^3^) | 3658.5 (3) |
| *Z* | 4 |
| Radiation type | Mo *K*α |
| μ (mm^-1^) | 1.24 |
| Crystal size (mm) | 0.41 × 0.26 × 0.05 |
|  | |
| Data collection | |
| Diffractometer | Bruker AXS D8 Quest diffractometer with PhotonII charge-integrating pixel array detector (CPAD) |
| Absorption correction | Multi-scan, *SADABS* 2016/2: Krause, L., Herbst-Irmer, R., Sheldrick G.M. & Stalke D., J. Appl. Cryst. 48 (2015) 3-10 |
| *T*_min_, *T*_max_ | 0.616, 0.747 |
| No. of measured, independent and observed [*I* > 2σ(*I*)] reflections | 33096, 10568, 7472 |
| *R*_int_ | 0.061 |
| (sin θ/λ)_max_ (Å^-1^) | 0.769 |
|  | |
| Refinement | |
| *R*[*F*^2^ > 2σ(*F*^2^)], *wR*(*F*^2^), *S* | 0.041, 0.085, 0.98 |
| No. of reflections | 10568 |
| No. of parameters | 438 |
| No. of restraints | 4 |
| H-atom treatment | H atoms treated by a mixture of independent and constrained refinement |
| Δρ_max_, Δρ_min_ (e Å^-3^) | 0.30, -0.39 |
| Absolute structure | Flack x determined using 2468 quotients [(I+)-(I-)]/[(I+)+(I-)] (Parsons, Flack and Wagner, Acta Cryst. B69 (2013) 249-259). |
| Absolute structure parameter | 0.010 (5) |

Computer programs: Apex3 v2019.1-0 (Bruker, 2019), *SAINT* V8.40A (Bruker, 2019), SHELXM (Sheldrick, 2008), *SHELXL2018*/3 (Sheldrick, 2015, 2018), SHELXLE Rev1117 (Hübschle *et al.*, 2011).

Table X: Experimental details for bedaquiline free base

|  | Bedaquiline free base |
| --- | --- |
| Crystal data | |
| Chemical formula | C_32_H_31_BrN_2_O_2_ |
| *M*_r_ | 555.50 |
| Crystal system, space group | Orthorhombic, *P*2_1_2_1_2_1_ |
| Temperature (K) | 150 |
| *a*, *b*, *c* (Å) | 11.1584 (8), 13.6425 (14), 36.061 (4) |
| *V* (Å^3^) | 5489.5 (9) |
| *Z* | 8 |
| Radiation type | Mo *K*α |
| μ (mm^-1^) | 1.53 |
| Crystal size (mm) | 0.21 × 0.13 × 0.05 |
|  | |
| Data collection | |
| Diffractometer | Bruker AXS D8 Quest diffractometer with PhotonII charge-integrating pixel array detector (CPAD) |
| Absorption correction | Multi-scan, *SADABS* 2016/2: Krause, L., Herbst-Irmer, R., Sheldrick G.M. & Stalke D., J. Appl. Cryst. 48 (2015) 3-10 |
| *T*_min_, *T*_max_ | 0.603, 0.747 |
| No. of measured, independent and observed [*I* > 2σ(*I*)] reflections | 66520, 17893, 12296 |
| *R*_int_ | 0.052 |
| (sin θ/λ)_max_ (Å^-1^) | 0.770 |
|  | |
| Refinement | |
| *R*[*F*^2^ > 2σ(*F*^2^)], *wR*(*F*^2^), *S* | 0.044, 0.111, 1.03 |
| No. of reflections | 17893 |
| No. of parameters | 675 |
| H-atom treatment | H-atom parameters constrained |
| Δρ_max_, Δρ_min_ (e Å^-3^) | 0.48, -0.58 |
| Absolute structure | Flack x determined using 4397 quotients [(I+)-(I-)]/[(I+)+(I-)] (Parsons, Flack and Wagner, Acta Cryst. B69 (2013) 249-259). |
| Absolute structure parameter | 0.034 (3) |

Computer programs: Apex3 v2019.1-0 (Bruker, 2019), *SAINT* V8.40A (Bruker, 2019), *SHELXS97* (Sheldrick, 2008), *SHELXL2018*/3 (Sheldrick, 2015, 2018), SHELXLE Rev1117 (Hübschle *et al.*, 2011).

Table XI: Experimental details for bedaquiline maleate 0.5hydrate single crystal

|  | Bedaquiline maleate 0.5hydrate single crystal |
| --- | --- |
| Crystal data | |
| Chemical formula | C_32_H_32_BrN_2_O_2_·C_4_H_3_O_4_·0.476(H_2_O) |
| *M*_r_ | 680.17 |
| Crystal system, space group | Monoclinic, *C*2 |
| Temperature (K) | 150 |
| *a*, *b*, *c* (Å) | 15.7469 (5), 13.2627 (4), 17.8602 (6) |
| β (°) | 106.3762 (13) |
| *V* (Å^3^) | 3578.7 (2) |
| *Z* | 4 |
| Radiation type | Cu *K*α |
| μ (mm^-1^) | 1.94 |
| Crystal size (mm) | 0.21 × 0.17 × 0.13 |
|  | |
| Data collection | |
| Diffractometer | Bruker AXS D8 Quest diffractometer with PhotonIII_C14 charge-integrating and photon counting pixel array detector |
| Absorption correction | Multi-scan, *SADABS* 2016/2: Krause, L., Herbst-Irmer, R., Sheldrick G.M. & Stalke D., J. Appl. Cryst. 48 (2015) 3-10 |
| *T*_min_, *T*_max_ | 0.646, 0.754 |
| No. of measured, independent and observed [*I* > 2σ(*I*)] reflections | 15060, 7003, 6474 |
| *R*_int_ | 0.040 |
| (sin θ/λ)_max_ (Å^-1^) | 0.638 |
|  | |
| Refinement | |
| *R*[*F*^2^ > 2σ(*F*^2^)], *wR*(*F*^2^), *S* | 0.041, 0.107, 1.08 |
| No. of reflections | 7003 |
| No. of parameters | 430 |
| No. of restraints | 8 |
| H-atom treatment | H atoms treated by a mixture of independent and constrained refinement |
| Δρ_max_, Δρ_min_ (e Å^-3^) | 0.35, -0.46 |
| Absolute structure | Flack x determined using 2633 quotients [(I+)-(I-)]/[(I+)+(I-)] (Parsons, Flack and Wagner, Acta Cryst. B69 (2013) 249-259). |
| Absolute structure parameter | 0.019 (8) |

Computer programs: Apex3 v2019.1-0 (Bruker, 2019), *SAINT* V8.40A (Bruker, 2019), *SHELXS97* (Sheldrick, 2008), *SHELXL2018*/3 (Sheldrick, 2015, 2018), SHELXLE Rev1143 (Hübschle *et al.*, 2011).

Table XII: experimental details for bedaquiline maleate THF solvate single crystal

|  | Bedaquiline maleate THF solvate single crystal |
| --- | --- |
| Crystal data | |
| Chemical formula | 2(C_32_H_32_BrN_2_O_2_)·2(C_4_H_3_O_4_)·3(C_4_H_8_O) |
| *M*_r_ | 1559.45 |
| Crystal system, space group | Monoclinic, *C*2 |
| Temperature (K) | 150 |
| *a*, *b*, *c* (Å) | 16.4119 (6), 13.5643 (6), 17.8475 (8) |
| β (°) | 107.318 (3) |
| *V* (Å^3^) | 3793.0 (3) |
| *Z* | 2 |
| Radiation type | Cu *K*α |
| μ (mm^-1^) | 1.92 |
| Crystal size (mm) | 0.33 × 0.19 × 0.16 |
|  | |
| Data collection | |
| Diffractometer | Bruker AXS D8 Quest diffractometer with PhotonIII_C14 charge-integrating and photon counting pixel array detector |
| Absorption correction | Multi-scan, *SADABS* 2016/2: Krause, L., Herbst-Irmer, R., Sheldrick G.M. & Stalke D., J. Appl. Cryst. 48 (2015) 3-10 |
| *T*_min_, *T*_max_ | 0.254, 0.391 |
| No. of measured, independent and observed [*I* > 2σ(*I*)] reflections | 18660, 7473, 6615 |
| *R*_int_ | 0.049 |
| (sin θ/λ)_max_ (Å^-1^) | 0.639 |
|  | |
| Refinement | |
| *R*[*F*^2^ > 2σ(*F*^2^)], *wR*(*F*^2^), *S* | 0.054, 0.127, 1.05 |
| No. of reflections | 7473 |
| No. of parameters | 551 |
| No. of restraints | 223 |
| H-atom treatment | H atoms treated by a mixture of independent and constrained refinement |
| Δρ_max_, Δρ_min_ (e Å^-3^) | 0.46, -0.52 |
| Absolute structure | Refined as an inversion twin. |
| Absolute structure parameter | 0.03 (3) |

Computer programs: Apex3 v2019.1-0 (Bruker, 2019), *SAINT* V8.40A (Bruker, 2019), *SHELXS97* (Sheldrick, 2008), *SHELXL2018*/3 (Sheldrick, 2015, 2018), SHELXLE Rev1143 (Hübschle *et al.*, 2011).

Table XIII: experimental details for bedaquiline besylate single crystal

|  | Bedaquiline besylate single crystal |
| --- | --- |
| Crystal data | |
| Chemical formula | C_42_H_47_BrN_2_O_7_S |
| *M*_r_ | 803.78 |
| Crystal system, space group | Orthorhombic, *P*2_1_2_1_2_1_ |
| Temperature (K) | 150 |
| *a*, *b*, *c* (Å) | 8.2545 (3), 17.1488 (8), 27.0285 (11) |
| *V* (Å^3^) | 3826.0 (3) |
| *Z* | 4 |
| Radiation type | Cu *K*α |
| μ (mm^-1^) | 2.41 |
| Crystal size (mm) | 0.08 × 0.07 × 0.01 |
|  | |
| Data collection | |
| Diffractometer | Bruker AXS D8 Quest diffractometer with PhotonIII_C14 charge-integrating and photon counting pixel array detector |
| Absorption correction | Multi-scan, *SADABS* 2016/2: Krause, L., Herbst-Irmer, R., Sheldrick G.M. & Stalke D., J. Appl. Cryst. 48 (2015) 3-10 |
| *T*_min_, *T*_max_ | 0.607, 0.754 |
| No. of measured, independent and observed [*I* > 2σ(*I*)] reflections | 22186, 7888, 5554 |
| *R*_int_ | 0.099 |
| (sin θ/λ)_max_ (Å^-1^) | 0.639 |
|  | |
| Refinement | |
| *R*[*F*^2^ > 2σ(*F*^2^)], *wR*(*F*^2^), *S* | 0.052, 0.113, 1.02 |
| No. of reflections | 7888 |
| No. of parameters | 489 |
| No. of restraints | 5 |
| H-atom treatment | H atoms treated by a mixture of independent and constrained refinement |
| Δρ_max_, Δρ_min_ (e Å^-3^) | 0.28, -0.45 |
| Absolute structure | Flack x determined using 1746 quotients [(I+)-(I-)]/[(I+)+(I-)] (Parsons, Flack and Wagner, Acta Cryst. B69 (2013) 249-259). |
| Absolute structure parameter | 0.02 (2) |

Computer programs: Apex3 v2019.1-0 (Bruker, 2019), *SAINT* V8.40A (Bruker, 2019), *SHELXS97* (Sheldrick, 2008), *SHELXL2018*/3 (Sheldrick, 2015, 2018), SHELXLE Rev1149 (Hübschle *et al.*, 2011).

Table XIV: Properties of the reagents and stages at which they were used in the study

| Reagent | Grade | Lo or Batch # | Manufacturer | Use in study |
| --- | --- | --- | --- | --- |
| 2-propanol | HPLC ≥ 99.9% | SHBM 1057 | Sigma Aldrich | Solvent for salt and polymorph screen |
| Acetic acid | HPLC grade | 46846 | Fischer Scientific, NJ | Salt former |
| Acetone | certified ACS | 183550 | Fischer Chemicals | Solvent for Salt and polymorph screen |
| Acetonitrile | for HPLC, super gradient reagent | 18J174008 | VWR (BDH) Chemicals | Salt and polymorph screen |
| Benzene Sulfonic acid | ACS grade, 94 % | Q07G032 | Alfa Aesar | Salt former |
| Benzoic acid | crystals, USP | KJJV | Mallinckrodt | Salt former |
| Dichloromethane | HPLC grade | 214321 | Avantor performance materials llc | Solvent for recovery of Bedaquiline base from Fumarate salt |
| Dimethylformamide (DMF) | HPLC grade | 19060194 | VWR (BDH) Chemicals | Solvent for polymorph screen |
| Ethyl Acetate | HPLC ≥ 99.5% | SHBL9034 | Sigma Aldrich | Solvent for polymorph screen |
| Ethyl Alcohol | ACS reagent ≥ 99.5% | SHBL9722 | Sigma Aldrich | Solvent for polymorph screen |
| Hexanes | ACS | 5189-04 | Mallinckrodt Chemicals | Solvent for polymorph screen |
| Hydrobromic acid | 48% | 251102 | VWR Chemicals | Salt former |
| Hydrochloric acid | ACS reagent, 37 % | MKCK1697 | Sigma Aldrich | Salt former |
| Lactic acid | ACS reagent, 85+ % solution in water | 15220PA | Sigma Aldrich | Salt former |
| Maleic acid | ACS grade, 98+ % | 50013961 | Beantown Chemical (BTC) | Salt former |
| Malic acid | ACS grade, 99+ % | 00513BJ | Sigma Aldrich | Salt former |
| Methane Sulfonic acid | ACS grade, 98+ % | X15E021 | Alfa Aesar | Salt former |
| Methanol | HPLC ≥ 99.9% | SHBL8762 | Sigma Aldrich | Solvent for polymorph screen |
| Methanolic Hydrochloric acid 1.25M | ACS | 1330542 42607182 | Fluka | Salt former |
| Propyl alcohol | HPLC grade | PX 1815-1 | MCB manufacturing chemists | Solvent for polymorph screen |
| Succinic acid | ACS grade, ≥ 99.0% | 099K0125 | Sigma Aldrich | Salt former |
| Tetrahydrofuran (THF) | HPLC ≥ 99.9% | 02158HE | Sigma Aldrich | Solvent for Salt and polymorph screen |

Table XV- Summary 1 of salt screen experiments (Purdue labs’ data)

| **Salt former** | **Acetone (SE)** | **Acetone (FE)** | **ACN (SE)** | **ACN (FE)** | **THF(FE)** | **IPA (SE)** | **IPA (heat)** |
| --- | --- | --- | --- | --- | --- | --- | --- |
| Acetic acid | Cryst. (low) |  | Amorph | Amorph |  |  |  |
| Benzene sulfonic acid | Amorph |  |  |  |  |  |  |
| Benzoic acid | Cryst. (SE),  (H_2_0 A),(Hex. A) | Cryst  Amor (H_2_0 A) | Cryst  Cryst (H_2_0 A) | Cryst  Cryst (H_2_0 A) |  |  |  |
| HCl acid | Cryst. (1:1, 2: 1, 3:1) | Cryst (low) |  |  |  | Amorph | Cryst (base) |
| Lactic acid |  |  | Cryst |  |  |  |  |
| Maleic acid | Amorph (1:1)  Amorph (1:2) | Cryst (1:2)  Amorph(2:1) | Amorph (1:1) | Amorph | Cryst. |  |  |
| Malic acid | Amorph (1:2) |  |  | Amorph |  |  |  |
| Succinic acid | Amorph (1:2) | Amorph(1:1) |  | Cryst (1:2)  Amorph(1:1) |  |  |  |
| Fumaric acid |  | Amorph |  |  |  | Cryst |  |
| Methanolic HCl | Cryst (free base) | Cryst (free base) | Cryst (free base) | Cryst (free base) |  |  |  |

H_2_0 A = Water used as anti-solvent (5:1, Solvent:water) Hex. A = hexane as anti-solvent Cryst = crystals formed

Amorph = Amorphous material Empty cells = no precipitates formed

SE = Slow Evaporation, FE= Fast Evaporation

Table XVI. Bedaquiline Small Scale Salt Screen Sample Information (improved Pharma data)

All experiments were FE from acetone with water as anti-solvent (~25 mg scale)

| **Sample** | **Counterion** | **Fast Evaporation**  **(Acetone)** | **Well Plate Fast Evaporation**  **(Acetone)** |
| --- | --- | --- | --- |
| RP1-15-1 | **Methanesulfonic acid** | Y | Y |
| RP1-15-2 | **Lactic acid** | Y | Y |
| RP1-15-3 | **Hydrochloric acid** | Y | Y |
| RP1-15-4 | **Acetic acid** | Y | Y |
| RP1-15-5 | **Maleic acid*** | Y | Y |
| RP1-15-6 | **Succinic acid*** | Y | Y |
| RP1-15-7 | **Malic acid*** | Y | Y |
| RP1-15-8 | **Benzoic acid** | Y | Y |
| RP1-15-9 | **Fumaric acid** | Y** | Y |
| RP1-15-10 | **Benzenesulfonic acid** | Y | Y |

*Set up as 2 : 1, Bedaquiline : counterion

**Initially clear gel-like

Table XVII. Bedaquiline Large Scale Salt Screen Sample Information (Improved Pharma data)

All experiments were SE (400 mg scale)

| **Sample** | **Counterion** | **Solvent** | **Precipitate** |
| --- | --- | --- | --- |
| RP1-42-2 | **Benzenesulfonic acid**  (400 mg) | THF | Y |
| RP1-42-3 | **Methanesulfonic acid**  (400 mg) | THF | Y |
| RP1-42-4 | **Maleic acid**  (400 mg) | THF | Y* |

Counterions were added directly to the Bedaquiline stock solution in scintillation vials; all experiments set up as 1 : 1, Bedaquiline : counterion.

*White crystals almost immediately

Table XVIII. Bedaquiline Medium Scale Salt Screen Sample Information (Improved Pharma data)

All experiments were SE or n-hexanes added as anti-solvent for SE (~50−100 mg scale)

| **Sample** | **Counterion** | **Solvent** | **Precipitate** | **Solvent** | **Precipitate** |
| --- | --- | --- | --- | --- | --- |
| RP1-21-1 | **Maleic acid**  (100 mg) | Acetone | N | THF | Y* |
| RP1-22-1 | **Methanesulfonic acid**  (50 mg) | IPA | N | − | N |
| RP1-22-2 | **Benzenesulfonic acid**  (75 mg) | THF | Y** | − | − |
| RP1-27-1 | **Malic acid**  (100 mg) | THF | N | Acetone | N |
| RP1-27-2 | **Succinic acid**  (100 mg) | THF | N | Acetone | N |
| RP1-27-3 | **Acetic acid**  (100 mg) | THF | N | Acetone | N |
| RP1-27-4 | **Hydrochloric acid**  (100 mg) | THF | N | Acetone | N |
| RP1-42-1 | **Succinic acid *****  (50 mg) | THF | Y |  |  |

For all experiments counterions were added directly to the Bedaquiline stock solution in scintillation vials. Subsequent SE experiments were done in 1 dram vials.

All experiments set up as 1 : 1, Bedaquiline : counterion.

*White/clear plates almost immediately

**White

***Solvent/anti-solvent

Table XIX- Summary 1 of salt screen experiments (Purdue labs’ data)

| **Solvent** | **Slow Evaporation** | **Fast Evaporation** | **Heating/ Cooling** | **Slurry (1week)** | **Antisolvent (water)** |
| --- | --- | --- | --- | --- | --- |
| Acetonitrile | BABQ XRPD | BABQ XRPD | BABQ XRPD | BABQ XRPD | BABQ XRPD |
| Acetone | BABQ XRPD | BABQ XRPD | ^^ | BABQ XRPD | BABQ XRPD |
| 2-Propanol | BABQ XRPD** |  | BABQ XRPD** | BQ XRPD* | BABQ XRPD** |
| Propyl alcohol |  |  | Amorphous^^^ | BABQ XRPD** | BABQ XRPD |
| Ethanol |  | BABQ XRPD | BABQ XRPD | BABQ XRPD | BABQ XRPD* |
| Methanol | BABQ XRPD | BABQ XRPD | BABQ XRPD | BABQ XRPD* | BABQ XRPD |
| Ethylacetate | BABQ XRPD | BABQ XRPD | Amorphous^^^ |  | BABQ XRPD |
| Hexane |  | BABQ XRPD | Amorphous^^^ | BABQ XRPD | BABQ XRPD |
| DMF |  |  | BABQ XRPD | BABQ XRPD |  |
| THF | ^ | ^ | ^ | ^ | ^ |

1 additional peak* High solubility, experiment not done^^

Amorphous^^^ →crystalline on heating @60^o^C. 24hrs

> 2 additional peaks** viscous liquid did not crystalize salt^

* Additional peaks were attributable to the free base

Table XX. Bedaquiline Slow and Fast Evaporation Experiments Sample Information. (Improved Pharma data)

| **Solvent** | **Sample** | **Slow Evaporation** | **XRPD Result** | **Sample** | **Fast Evaporation** | **XRPD Result** |
| --- | --- | --- | --- | --- | --- | --- |
| MeOH | RP1-79-4 | Oily/white ppt | Amorphous | RP1-88-1 | White, glassy ppt | Amorphous |
| Propanol | RP1-79-5 | Clear/white ppt | Amorphous | RP1-88-2 | Clear, oily ppt | Amorphous |
| Acetone | RP1-79-6 | Clear, oily ppt | Amorphous | RP1-88-3 | White, glassy ppt, blades? | Crystalline – refines to water solvate |
| EtOH | RP1-79-7 | Clear ppt | – | RP1-88-4 | Clear, oily ppt, some white ppt | – |
| ACN | RP1-79-8 | White ppt, blades? | Crystalline – refines to water solvate + pk @ 12.2 °2-theta | RP1-88-5 | White ppt, some blades? | Crystalline – refines to water solvate |
| IPA | RP1-79-9 | Clear, oily ppt | Amorphous | RP1-88-6 | Clear, oily ppt | Amorphous |
| EtAC | RP1-79-10 | White ppt, blades? | Crystalline – refines to water solvate + pk @ 12.2 °2-theta | RP1-88-7 | White ppt, many blades? | Crystalline – refines to water solvate |
| TFE | RP1-87-1 | Clear, oily ppt | Amorphous | RP1-88-8 | Clear, oily ppt | Amorphous |

Table XXI. Bedaquiline Maleate Solvent/Anti-Solvent Experiments Sample Information.

Experiments were either fast evaporation or slow and fast evaporation following anti-solvent addition

| **Sample** | **Solvent** | **Anti-Solvent** | **Observations** | **XRPD Result** |
| --- | --- | --- | --- | --- |
| RP1-87-2 | DMF | Water | Ppt quickly; clear, glassy ppt** | Could not isolate |
| RP1-87-3 | Trifluoroethanol | Water | White ppt** | Amorphous |
| RP1-90-2 | Methanol | Water | White, glassy ppt** | Amorphous |
| RP1-90-3 | n-propanol | Water | No ppt; FE; white ppt* | crystalline- refines to mostly free base (93.7%) + water solvate + amorphous |
| RP1-90-4 | Acetone | Water | White ppt, blades, glassy** | crystalline- refines to mostly free base (87.2%) + water solvate+ amorphous |
| RP1-90-5 | Acetonitrile | Water | White ppt, blades, glassy** | crystalline- refines to mostly free base (89.3%) + water solvate+ amorphous |
| RP1-90-6 | Ethyl acetate | Water | White ppt ** | Amorphous |
| RP1-90-7 | Ethanol | Water | FE; white ppt* | Amorphous |
| RP1-90-8 | n-propanol | n-hexanes | No ppt; FE; oily, gummy ppt* | Could not isolate |
| RP1-91-6 | Ethanol | n-hexanes | Didn’t dissolve; oily, gummy ppt** | Could not isolate |
| RP1-91-7 | Isopropyl alcohol | n-hexanes | Didn’t dissolve; oily, gummy ppt** | Could not isolate |
| RP1-91-8 | Acetone | n-hexanes | Didn’t dissolve; oily, gummy ppt** | Amorphous |
| RP1-91-9 | Ethyl acetate | n-hexanes | Didn’t dissolve; oily, gummy ppt** | Amorphous |

*FE (fast evaporation)

**SE (slow evaporation) / FE (fast evaporation)

Table XXII. Sorption experiments for bedaquiline salts.

| **Sample** | **Counterion** | **Initial weight**  **(mg)** | **75% RH**  **weight gain (%)** | **Initial weight**  **(mg)** | **0% RH weight loss (%)*** |
| --- | --- | --- | --- | --- | --- |
| RP1-42-2 | **Benzenesulfonic acid**  (400 mg) | 19.0 | 66.8** | 19.0 | 12.6*** |
| RP1-42-3 | **Methanesulfonic acid**  (400 mg) | 15.7 | 80.0** | 15.0 | 6.4*** |
| RP1-42-4 | **Maleic acid**  (400 mg) | 24.2 | <0.1**** | 24.0 | - |
| BABQ  IPA/water  (Purdue lab) | **Benzoic acid** | 18.7 | <0.1**** | 20.7 | <0.1**** |
| BQ HCl  Acetone SE  (Purdue lab) | **HCl** | 2.1 | <0.1*** | 2.7 | <0.1*** |
|  |  |  |  |  |  |

* weight loss after 75% RH exposure

** 9 days exposure

*** 25 days exposure

**** 41 days exposure
